# Comparison of Sirtuin 3 Levels in ALS and Huntington’s Disease—Differential Effects in Human Tissue Samples vs. Transgenic Mouse Models

**DOI:** 10.3389/fnmol.2017.00156

**Published:** 2017-05-26

**Authors:** Eva Buck, Hanna Bayer, Katrin S. Lindenberg, Johannes Hanselmann, Noemi Pasquarelli, Albert C. Ludolph, Patrick Weydt, Anke Witting

**Affiliations:** ^1^Department of Neurology, Ulm UniversityUlm, Germany; ^2^Department of Neurodegenerative Disorders and Gerontopsychiatry, Bonn UniversityBonn, Germany

**Keywords:** amyotrophic lateral sclerosis, Huntington’s disease, Sirt3, PGC-1α, mitochondria, SOD(G93A), R6/2, human tissue

## Abstract

Neurodegenerative diseases are characterized by distinct patterns of neuronal loss. In amyotrophic lateral sclerosis (ALS) upper and lower motoneurons degenerate whereas in Huntington’s disease (HD) medium spiny neurons in the striatum are preferentially affected. Despite these differences the pathophysiological mechanisms and risk factors are remarkably similar. In addition, non-neuronal features, such as weight loss implicate a dysregulation in energy metabolism. Mammalian sirtuins, especially the mitochondrial NAD+ dependent sirtuin 3 (SIRT3), regulate mitochondrial function and aging processes. SIRT3 expression depends on the activity of the metabolic master regulator peroxisome proliferator-activated receptor gamma coactivator 1-alpha (PGC-1α), a modifier of ALS and HD in patients and model organisms. This prompted us to systematically probe Sirt3 mRNA and protein levels in mouse models of ALS and HD and to correlate these with patient tissue levels. We found a selective reduction of *Sirt3* mRNA levels and function in the cervical spinal cord of end-stage ALS mice (superoxide dismutase 1, SOD1^G93A^). In sharp contrast, a tendency to increased *Sirt3* mRNA levels was found in the striatum in HD mice (R6/2). Cultured primary neurons express the highest levels of *Sirt3* mRNA. In primary cells from PGC-1α knock-out (KO) mice the *Sirt3* mRNA levels were highest in astrocytes. In human *post mortem* tissue increased mRNA and protein levels of Sirt3 were found in the spinal cord in ALS, while Sirt3 levels were unchanged in the human HD striatum. Based on these findings we conclude that SIRT3 mediates the different effects of PGC-1α during the course of transgenic (tg) ALS and HD and in the human conditions only partial aspects Sirt3 dysregulation manifest.

## Introduction

Neurodegenerative diseases, including amyotrophic lateral sclerosis (ALS) and Huntington’s disease (HD) show distinct patterns of progressive neuronal cell loss. In ALS upper and lower motoneurons are lost, while in HD medium spiny neurons of the striatum are most prominently affected (Przedborski et al., 2003). Despite the differences in the vulnerable neuron populations, both diseases show similar cellular and molecular mechanisms including inflammation (Heneka et al., 2014), mitochondrial dysfunction (Carri et al., 2017; Polyzos and McMurray, 2017) and oxidative stress (Lin and Beal, 2006).

ALS and HD patients also show hypermetabolic features (Dupuis et al., 2004, 2011; Aziz et al., 2010; Tefera and Borges, 2017). These include loss of body weight, in spite of increased food intake (Djousse et al., 2002; Dupuis et al., 2011), and suggest an increase in energy expenditure (Dupuis et al., 2004, 2011; Aziz et al., 2008). The mitochondrial changes in ALS and HD are reminiscent of mitochondrial alterations during aging including the decreased capacity to cope with oxidative stress (Mecocci et al., 1993) and an increased rate of mitochondrial DNA (mtDNA) mutations (Lin and Beal, 2006).

Peroxisome proliferator-activated receptor gamma coactivator 1-alpha (PGC-1α) links mitochondrial and transcriptional dysfunction in aging and neurodegeneration (Rona-Voros and Weydt, 2010). PGC-1α is a disease modifier in human ALS and HD, and this effect is in part sex-dependent (Eschbach et al., 2013; Weydt et al., 2014). The underlying mechanisms are only beginning to be understood. Multiple promoters and alternative splicing of the *Ppargc1a* gene allow for a versatile regulation of the system (Martínez-Redondo et al., 2015). In ALS the situation is particularly complex, as different isoforms are affected differently in different tissues (Bayer et al., 2017). In ALS the canonical and central nervous system (CNS)-specific PGC-1α system is downregulated in disease relevant tissues of the SOD1(G93A) mouse model (Thau et al., 2012; Bayer et al., 2017). In HD both the canonical and CNS-specific PGC-1α signaling is inhibited (Cui et al., 2006; Weydt et al., 2006; Kim et al., 2010). In addition, PGC-1α knock-out (KO) mice show neurodegenerative changes in the striatum resembling HD pathology (Lin et al., 2004; Rona-Voros and Weydt, 2010). PGC-1α dysfunction also mediates pathological aging (Sahin and DePinho, 2012). In the brain PGC-1α counteracts oxidative stress and protein aggregation (Tsunemi et al., 2012; Bayer et al., 2017). At the transcriptional level PGC-1α regulates the expression of sirtuin 3 (SIRT3), itself an important mitochondrial regulatory protein, via the estrogen related receptor alpha (ERRα; Zhang et al., 2016).

Mitochondrial sirtuins, especially SIRT3, link aging and mitochondrial function. The deacetylase SIRT3 is one of the seven mammalian sirtuins, localized to the mitochondria (Michishita et al., 2005). SIRT3 regulates mitochondrial reactive oxygen species (ROS) levels by deacetylating and activating the superoxide dismutase 2 (SOD2; Qiu et al., 2010; Tao et al., 2010; Chen et al., 2011). SIRT3 is also emerging as a regulator of key mitochondrial pathways (Lombard et al., 2007; Bause and Haigis, 2013) such as fatty acid oxidation (Hirschey et al., 2010), mitochondrial dynamics (Samant et al., 2014) and glucose utilization (Huynh et al., 2015).

Inflammatory processes in human macrophages are also mediated by SIRT3 (Traba et al., 2015) and inflammation is another shared feature of neurodegenerative diseases, including ALS and HD (Heneka et al., 2014). Inflammation is sensitive to metabolic changes, for example in macrophages, when aerobic glycolysis enables the secretory and respiratory bursts (Newsholme et al., 1986; Chawla et al., 2011). Furthermore, dysfunctional mitochondria result in an inhibition of the anti-inflammatory M2 response of microglia (Ferger et al., 2010).

The aim of this study was to investigate SIRT3 expression in ALS and HD. In both diseases SIRT3 expression and function was investigated in the CNS of mouse models of ALS (SOD1(G93A)) and HD (R6/2) and in human post mortem CNS tissue.

## Materials and Methods

### Human Biomaterial

Human biomaterial was handled according to appropriate approval and procedures. Autopsy material was obtained from the New York Brain Bank (Dr. Vonsattel) and the local brain bank with informed written consent and approved by the national medical ethical review boards in accordance with the world medical association Declaration of Helsinki. Clinical data of ALS and HD autopsy material is provided, showing the age, post mortem time and gender (Supplementary Table S1). Tissues of ALS patients derived from end-stage patients. One sample carried a SOD1 mutation but single nucleotide polymorphisms (SNPs) in the PGC-1α locus were not analyzed. The neuropathological stage of the HD autopsy samples included stage 1–4 cases with a mean of 2.9 (SD ± 0.9).

### Mouse Models

The SOD1 transgenic (tg) B6SJL-Tg(SOD1*G93A)1Gur/J mouse model for ALS, the PGC-1α KO B6.129-Ppargc1a^tm1Brsp^/J mouse model, the R6/2 HD mouse model B6CBATg(HDexon1)62Gpb/1J and the C57BL/6J mouse were used in this study. All mice were purchased from The Jackson Laboratory (USA). Only male mice were used for the experiments. Mice were bred under approved conditions of the animal facility of Ulm University and the Regierungspräsidium Tübingen (Reg. C. 0177). After 60 days (pre-onset), 100 days (onset) and about 130 days (end-stage) SOD1(G93A) tg and wild type (wt) littermates were sacrificed. End-stage was determined as either the inability to rise immediately after being placed on the side or as a weight loss of more than 20% (in respect to the highest body weight before onset of symptoms), defined by the Animal Ethics committee of the Regional Steering Committee Tübingen. PGC-1α KO mice were sacrificed at 90 days of age. R6/2 mice were used for experiments at the age of 30 days, 60 days and 90 days. Natively frozen tissue of male SIRT3 KO mice (129-*Sirt3^tm1.1Fwa^*/J) with the age of 30–60 days was obtained from the Jackson Laboratory (Bar Harbor, ME, USA).

Genotyping of the SOD1(G93A) and PGC-1α KO mice was performed according to the Jackson Laboratory standard PCR protocol^1^. R6/2 mice were genotyped as described previously (Mangiarini et al., 1996).

### Primary Cell Culture

Primary cells were prepared from C57BL/6J mice, B6SJL-Tg(SOD1*G93A)1Gur/J mice and B6.129-Ppargc1a^tm1Brsp^/J mice. Primary cultures of oligodendrocyte precursor cells (P0–2 mice) were prepared according to a protocol published previously (O’Meara et al., 2011). Neurons (E15), microglia (P0–5) and astrocytes (P0–5) were prepared as previously described (Wiesner et al., 2013). Substances and solutions were from Gibco™ or Sigma-Aldrich. In brief for neurons, astrocytes and microglia, forebrains were digested and dissociated. All cells were seeded in supplemented dulbecco’s modified eagle medium (DMEM; #31966). Neurons grew on 6-well plates with 6 × 10^5^ cells per well. Media was exchanged to supplemented Neurobasal (#21103) at day 1.5 at day 7. Neurons were stimulated after 14 days *in vitro*. After 7–10 days in culture, microglia were seeded on 6-well plates with 6 × 10^5^ cells per well for stimulation. Astrocytes were seeded on 6-well plates with 1 × 10^5^ cells per well for stimulation.

### Cell Lines

The murine neuroblastoma cell line Neuro-2a (DSMZ, #ACC148) grew in DMEM (#31966) supplemented with 10% heat-inactivated feta bovine serum (FBS), 100 U/ml penicillin and 100 μg/ml streptomycin.

### Luciferase Assay

For luciferase reporter assays Neuro-2a cells were seeded at a density of 10,000 cells per well of a poly-L-ornithine coated 96-well plate (#655098 Greiner, Frickenhausen, Germany) 24 h prior to lipofectamine transfection in DMEM (#31966) supplemented with 10% heat-inactivated FBS, 100 U/ml penicillin and 100 μg/ml streptomycin. The plasmid containing a firefly luciferase gene under the control of the SIRT3 promoter was generously provided by Marcia Haigis (Harvard Medical School, Boston, MA, USA; Satterstrom and Haigis, 2014). Cells were transfected with the renilla containing plasmid for normalization of transfection efficiency and the SIRT3-firefly construct. In addition, different isoforms of PGC-1α on a pCI backbone were added including canonical wt PGC-1α, the brain specific B4-PGC-1α or the brain specific B5-PGC-1α plasmid as described previously (Bayer et al., 2017). The PGC-1α constructs were generously provided by Wolfang Patsch. The Dual Glo luciferase assay (E2940, Promega, Madison, WI, USA) was performed 48 h after transfection in accordance with the manufacturer’s instruction. All luciferase experiments were measured in triplicates (#2030 VICTOR™ X3, Perkin Elmer, Waltham, MA, USA). The relative light units (RLU) measured of the firefly activity were normalized to the RLU of the renilla and shown in percent of the canonical PGC-1α.

### qPCR

RNA was isolated according to the manufacturer’s protocol from tissue samples (RNeasy Plus Universal Mini Kit, Qiagen, Venlo, Netherlands) and cell pellets (RNeasy Plus Mini kit, Qiagen, Venlo, Netherlands), whereas a the RNeasy Plus Micro kit (#73404, Qiagen, Venlo, Netherlands) was used for cell numbers below 5 × 10^5^, including stimulated microglia and neurons. One microgram of RNA was transcribed to cDNA using the iScript cDNA synthesis kit (Bio-Rad, Hercules, CA, USA). The quantitative real-time PCR (qPCR) was performed in duplicates on a CFX348 Touch Real-Time Detection System with iQ™SYBR® Green Supermix (Bio-Rad, Hercules, CA, USA). A standard curve was determined for every target to analyze the primer efficiency. The program consisted of the following steps: 95°C for 3 min, 40 cycles at 95°C for 15 s and a primer specific annealing temperature for 15 s, finally a 60°C to 95°C melting curve at increments with steps of 0.5°C every 5 s was determined. Data was analyzed by the Bio-Rad CFX manager (Bio-Rad, Hercules, CA, USA) software. At least two reference genes with a stable expression (mean CV <0.25, mean M value <0.5) were necessary for data analysis. Primer sequences of the targets examined and the reference genes used are indicated in Supplementary Table S2 for murine targets and in Supplementary Table S3 for human targets.

### Mitochondrial DNA Copy Number

Genomic and mtDNA were isolated from cell pellets with the Puregene Core Kit B (1042608, Qiagen, Venlo, Netherlands) according to the manufacturer’s instructions. The levels of the nuclear encoded single copy gene beta-2 microglobulin (B2M) and the stable region of the mitochondrial genome, the displacement loop (Dloop) were determined in triplicates by qPCR. 8 ng DNA were used per sample in a reaction mix, including iQ™SYBR® Green Supermix (Bio-Rad, Hercules, CA, USA), the respective primers (Supplementary Table S4) and water. The program run for 10 min at 95°C, 40 cycles of 10 s at 95°C, 15 s at 61°C, 20 s at 72°C, finally a 60–95°C melting curve was determined by increments of 1°C every 5 s (Hering et al., 2015). The ratio of the mitochondrial to half of the nuclear DNA copy number represents the relative mtDNA abundance.

### Western Blot

Lysates for western blot were prepared in RIPA (150 mM NaCl, 50 mM tris pH 7–8, 2% NP-40, 1% Na-Deoxycholat, 0.2% SDS) and protein concentration was determined using the bicinchoninic acid (BCA) Protein Assay Kit (BCA1, B9643). The appropriate amount of protein was boiled for 10 min at 95°C in Laemmli buffer (62.5 mM tris pH 6.8, 2% SDS, 10% glycerol, 0.002% bromphenol blue, 5% β-mercaptoethanol). Samples were separated on a 13% acrylamide gel and wet blotted on a polyvinylidene fluoride (PVDF) membrane. After protein transfer all proteins were stained by MemCode (24585, Thermo Fisher Scientific, Waltham, MA, USA) as loading control and reference for analysis. Primary antibodies against SIRT3 (#5490, Cell signaling, Danvers, MA, USA), SOD2 (ab13533, abcam, Cambridge, UK), SOD2 Ac-K122 (generously provided by David R. Gius, Robert H Lurie Medical Research Center, Chicago, IL, USA) and citrate synthase (CS; ab129095, abcam, Cambridge, UK) were applied to the membrane in 3% bovine serum albumin. Respective horseradish peroxidase (HRP) conjugated secondary antibodies (goat anti mouse HRP conjugated, 172–1011; goat anti rabbit HRP conjugated, 172–1019; Bio-Rad, Hercules, CA, USA) were used and the chemiluminescent signal was detected with Luminata Forte Western HRP substrate (WBLUF0500, Merck Millipore, Billerica, MA, USA) using the LAS (GE Healthcare, Chicago, IL, USA).

### Statistical Analysis

Statistical analysis of the data was performed using GraphPad prism 6. Two samples were compared for statistical significance by the unpaired student’s *t*-test for normally distributed data, which was tested by the Kolmogorov-Smirnow test. For small sample sizes and non Gaussian distributed data the nonparametric Mann-Whitney test was applied to compare two samples. For comparison of a group of samples the one-way ANOVA test was chosen with the Tukey’s multiple comparison test as parametric test and the Kruskal-Wallis test followed by Dunn’s correction as posttest was chosen for nonparametric data. To compare several groups the two-way ANOVA test with Tukey’s posttest for multiple comparison was chosen for the comparison of different genotypes and ages. To compare either differences between the genotypes or ages, the two-way ANOVA analysis was followed by Sidak’s multiple comparison test. Significance is indicated by ns = *P* > 0.05, *P ≤ 0.05, **P ≤ 0.01, ***P ≤ 0.001, ****P ≤ 0.0001. Data are shown as mean ± SEM.

## Results

### *Sirt3* Levels Decrease Specifically in Affected Brain Regions of the ALS SOD1(G93A) Mouse Model

**Figure 1:**
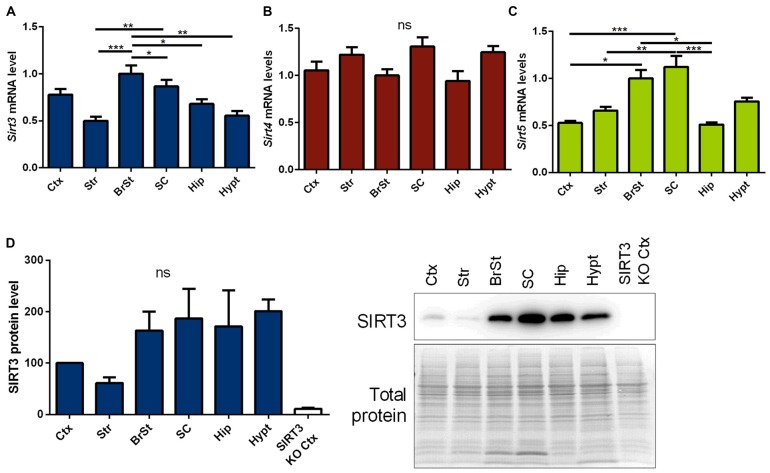
**Distribution of mitochondrial sirtuins in different regions of the murine central nervous system (CNS).** Cortex (Ctx), striatum (Str), brain stem (BrSt), spinal cord (SC), hippocampus (Hip) and hypothalamus (hypt) of 60 day old B6SJL mice were compared according to their *sirtuin 3* (*Sirt3*) **(A)**, *sirtuin 4* (*Sirt4*) **(B)** and *sirtuin 5* (*Sirt5*) **(C)** mRNA levels. mRNA levels were quantified by quantitative real-time PCR (qPCR) and normalized to TATA box binding protein (*Tbp*) and RNA polymerase 2 (*Polr2a*). Data are shown relative to BrSt levels (*n* = 5–6). One-way ANOVA was used for statistical analysis followed by Tukey’s multiple comparison test. **(D)** The tissue comparison was performed on SIRT3 protein level as well using 100 day old B6SJL mice, including cortex of SIRT3 knock-out (KO; 129-*Sirt3*^tm1.1Fwa^/J) mice (*n* = 3–6). Protein levels were normalized to total protein amount determined by MemCode staining. The nonparametric Kruskal-Wallis test was used followed by Dunn’s correction. Data are presented as mean ± SEM.ns = *P* > 0.05, *P ≤ 0.05, **P ≤ 0.01, ***P ≤ 0.001.

First we measured Sirt3 mRNA and protein levels, in informative brain regions of wt mice. These were particularly high in the brain stem and spinal cord, two regions prominently affected in ALS (Figures 1A,D, Supplementary Figure 5). In contrast, *sirtuin 4* (S*irt4*) mRNA levels were equally expressed across the different brain regions (Figure 1B), while *sirtuin 5* (*Sirt5*) mRNA expression showed a region specific distribution similar to *Sirt3* (Figure 1C).

**Figure 2:**
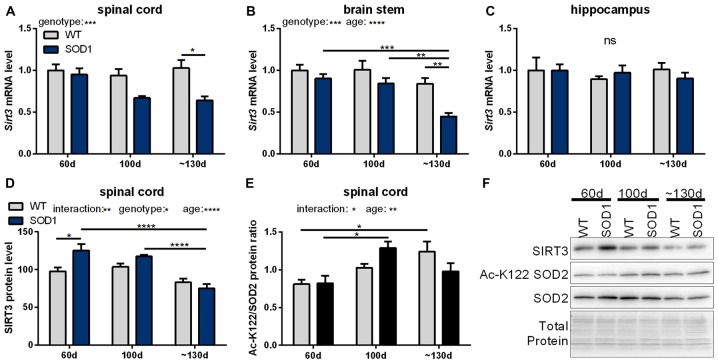
**Sirtuin 3 (SIRT3) levels and function in SOD1(G93A) mice decrease in affected tissues during the course of disease.** *Sirt3* mRNA levels were determined in B6SJL-Tg(SOD1*G93A)1Gur/J (SOD1(G93A)) mice during the course of disease at 60 days (preonset), 100 days (onset) and at about 130 days (endstage) in the disease affected brain regions spinal cord **(A)**, the brain stem **(B)** and the control region hippocampus **(C)**, *n* = 5–8. mRNA levels were quantified by qPCR and normalized to TATA box binding protein (*Tbp*) and RNA polymerase 2 (*Polr2a*) in **(A)**, to β-actin (*Actb*) and ribosomal protein large P0 (*Rplp0*) in **(B)** and to *Tbp*, *Polr2a* and *Actb* in **(C)**, relative to mean of wild type (wt) 60 old mice. Protein levels of SIRT3 decreased in SOD1(G93A) mice with age in the spinal cord **(D)**. SIRT3 function was determined by the acetylation status of lysine 122 (K122) of the superoxide dismutase 2 (SOD2), which is deacetylated by SIRT3. A high ratio of the acetylated SOD2 levels to the total SOD2 amount indicates decreased SIRT3 activity **(E)**, *n* = 6. Protein levels were measured by western blot and normalized to total protein amount determined by MemCode staining. Representative bands are shown in **(F)**. The grouped data was analyzed using the two-way ANOVA test followed by Tukey’s multiple comparison test. Data are presented as mean ± SEM. ns = *P* > 0.05, *P ≤ 0.05, **P ≤ 0.01, ***P ≤ 0.001, ****P ≤ 0.0001.

In a next step, mRNA levels of mitochondrial sirtuins in the spinal cord and brain stem were investigated during the disease progression in SOD1(G93A) mice. In end-stage mice *Sirt3* mRNA levels were significantly decreased in the spinal cord (Figure 2A) and brain stem (Figure 2B) compared to wt mice. In unaffected tissues of the CNS such as the hippocampus *Sirt3* mRNA levels did not change (Figure 2C). The decrease in *Sirt3* mRNA levels in the affected tissue was specific as the expression of the other mitochondrial sirtuins did not change during the disease progression, except for *Sirt5* mRNA levels in the brain stem (Supplementary Table S5).

Protein levels of SIRT3 were determined in tissue homogenates of the spinal cord of SOD1(G93A) animals. In 60 days old mice SIRT3 protein levels were higher in SOD1(G93A) tg animals compared to wt littermates (Figure 2D). SIRT3 protein levels decreased in the spinal cord of SOD1(G93A) tg animals in an age-dependent manner, dropping to wt levels at endstage (Figure 2D). One of the targets of SIRT3 is the ROS detoxifying enzyme SOD2, which is deacetylated by SIRT3 on lysine residue 122 and thereby activated (Tao et al., 2010). To investigate the function of SIRT3, we quantified the acetylation status of SOD2 by an acetylation specific antibody against acetylated lysine 122 (K122) of SOD2. The ratio of the acetylated to total SOD2 in the spinal cord of SOD1(G93A) tg mice and in littermate controls increased with time indicating a general age dependent decline of SIRT3 activity (Figure 2E). In SOD1(G93A) mice this age dependent decline in SIRT3 function was accelerated. Of note, we detected decreased protein levels of SOD2 in the spinal cord of diseased SOD1(G93A) tg animals (Figure 2F, Supplementary Figure 6).

### *Sirt3* Levels Are Highest in Neurons Independent of the SOD1(G93A) Mutation Status

**Figure 3:**
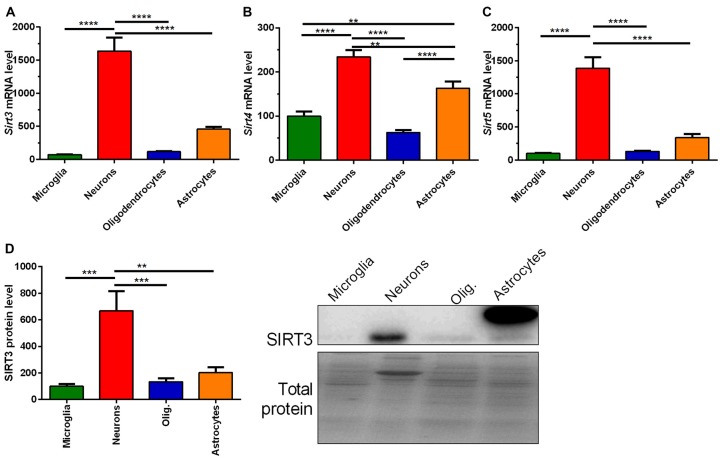
**Mitochondrial sirtuin levels are highest in primary cultured neurons compared to microglia, astrocytes and oligodendrocytes.** mRNA levels of *Sirt3* **(A)**, *Sirt4* **(B)** and *Sirt5* **(C)** were determined in primary cultured microglia, neurons, oligodendrocytes and astrocytes of C57BL/6J mice using qPCR. Data was normalized to TATA box binding protein (*Tbp*) and RNA polymerase 2 (*Polr2a*). Protein levels of SIRT3 were determined by western blot **(D)**. Protein data was normalized to the total protein amount determined by MemCode staining. Data were analyzed by the one-way ANOVA followed by Tukey’s multiple comparison test, *n* = 5–8. Data are shown as mean ± SEM. ns = *P* > 0.05, **P ≤ 0.01, ***P ≤ 0.001, ****P ≤ 0.0001.

Next we addressed whether the reduced SIRT3 expression is due to the loss of neurons. To answer this question we characterized the mitochondrial sirtuins in primary neurons and glia cells. All mitochondrial sirtuin mRNA levels were highest in neurons, and second highest in astrocytes (Figures 3A–C). The lowest levels of mitochondrial sirtuins were found in microglia and oligodendrocytes (Figures 3A–C). This result was confirmed on protein level for SIRT3 (Figure 3D, Supplementary Figure 7). These results were obtained by analyzing the SIRT3 band at a height of 28 kDa, which is represented by the lower band on the membranes. For astrocytes an additional band was obtained at about 36 kDa. The high levels of mitochondrial sirtuins in neurons could be either due to an elevated mitochondrial mass or due to an increased number of mitochondrial sirtuins per mitochondrial mass. We analyzed the expression of SIRT3 in relation to the mitochondrial mass by quantifying the mitochondrial matrix protein CS and the relative mtDNA abundance. CS protein levels were highest in neurons compared to other cell types (Supplementary Figure S1A), arguing for an increased mitochondrial mass in neurons. The relative mtDNA abundance also indicated high levels of mitochondrial mass in neurons, but the levels were also elevated in astrocytes (Supplementary Figure S1B). The ratio of SIRT3 protein levels per CS protein level showed a significantly increased ratio for neurons (Supplementary Figure S1C), indicating that neurons show both, increased mitochondrial mass *and* increased levels of mitochondrial sirtuins per mitochondrial mass. The ratio of SIRT3 protein levels per relative mtDNA abundance supported highest levels for SIRT3 per mitochondrion in neurons, but significance was reached only compared to astrocytes (Supplementary Figure S1D). We next investigated whether the SOD1 mutation has a direct effect on the *Sirt3* mRNA expression. Examination of different primary cultured cell types derived from SOD1(G93A) tg and wt animals did not show any differences according to the genotypes (Supplementary Figures S2A–D).

### Inflammatory Stimulation with LPS Reduces Mitochondrial Sirtuin Levels in Glia Cells

**Figure 4:**
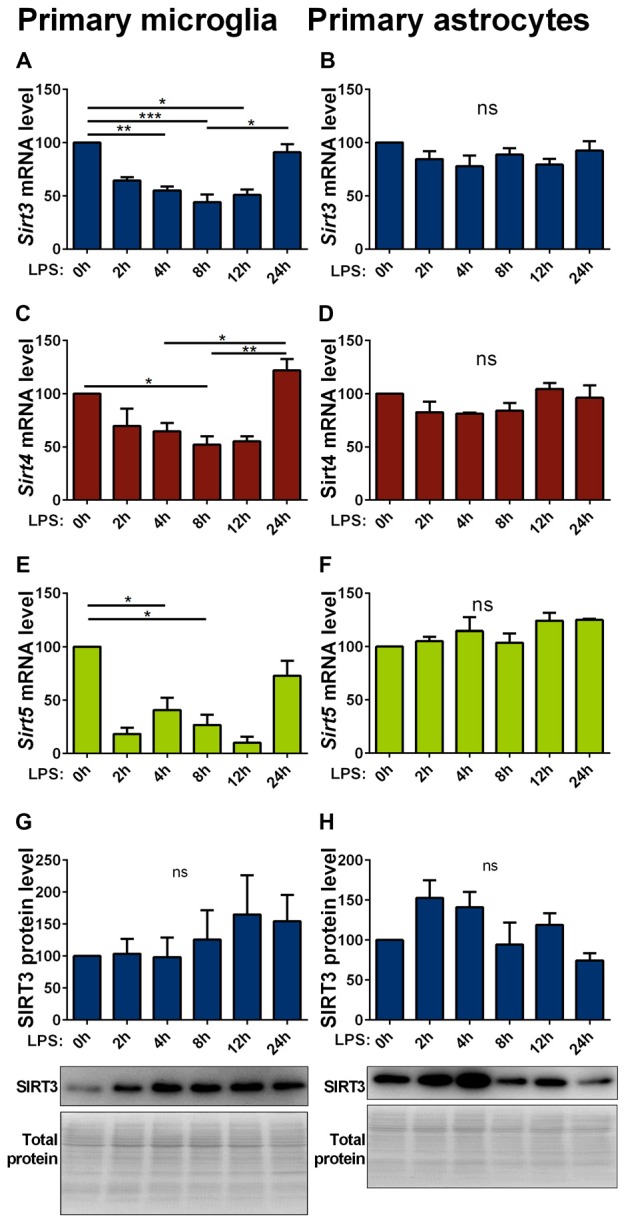
**Lipopolysaccharide (LPS) stimulation decreases Sirt3 mRNA levels in primary cultured microglia but not in astrocytes.** Cultured primary microglia **(A,C,E,G)** and astrocytes **(B,D,F,H)** were treated with 1 μg/ml LPS for 0, 2, 4, 8, 12 and 24 h. *Sirt3* **(A, B)**, *Sirt4* **(C,D)** and *Sirt5* **(E,F)** mRNA levels were determined by qPCR and normalized to TATA box binding protein (*Tbp*), RNA polymerase 2 (*Polr2a*) for microglia and additionally to β-actin (*Actb*) for astrocytes. SIRT3 protein levels were determined by western blot and normalized to the total protein amount determined by MemCode staining **(G,H)**. Data were normalized to the untreated control and analyzed by the nonparametric Kruskal-Wallis test, followed by Dunn’s multiple comparison test with *n* = 2–8 for microglia and *n* = 3–4 for astorcytes. Data are shown as mean ± SEM. ns = *P* > 0.05, *P ≤ 0.05, **P ≤ 0.01, ***P ≤ 0.001.

Inflammatory stimuli affect SIRT3 expression in macrophages (Liu et al., 2015; Traba et al., 2015). As inflammatory responses are an integral part of neurodegenerative diseases, we investigated their effect on mitochondrial sirtuin expression in microglia cells and astrocytes. In primary microglia lipopolysaccharide (LPS) stimulation decreased the mRNA expression of all investigated mitochondrial sirtuins (Figures 4A,C,E). The same treatment on astrocytes did not have any effect on the sirtuin expression (Figures 4B,D,F). The protein levels in both cell types were overall more variable and showed trend towards an increase in microglia cells, but did not formally reach statistical significance (Figures 4G,H, Supplementary Figures 8, 9). The SOD1(G93A) mutation had no effect on the LPS induced reduction of mitochondrial sirtuin levels in primary microglia cells (Supplementary Figures S3A–C).

### PGC-1α Changes the Cellular Expression Pattern of *Sirt3*

**Figure 5:**
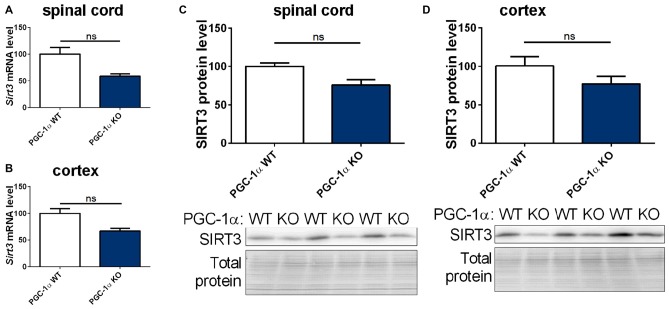
**Sirt3 levels show a decreasing tendency in the spinal cord and cortex of peroxisome proliferative-activated receptor gamma coactivator 1 alpha (PGC-1α) KO mice.** *Sirt3* **(A,B)** mRNA levels were determined by qPCR in the spinal cord **(A)** and cortex **(B)** of 90 day old B6.129-Ppargc1a^tm1Brsp^/J (PGC-1α KO) mice and respective controls. mRNA levels were normalized to TATA box binding protein (*Tbp*), RNA polymerase 2 (*Polr2a*) and β-actin (*Actb*). Levels are shown in percent of the wt control. Protein levels of SIRT3 were determined in the spinal cord **(C)** and cotex **(D)** by western blot and data were normalized to total protein amount determined by MemCode staining. The nonparametric Mann-Whitney test was used for analysis with *n* = 3. Data are shown as mean ± SEM.

The PGC-1α system is closely linked to the sirtuin system (Canto and Auwerx, 2009; Kong et al., 2010). We therefore quantified the mRNA and protein levels of mitochondrial sirtuins in CNS tissue of PGC-1α wt and KO mice. We found a trend towards decreased levels of *Sirt3, 4* and *5* mRNA and SIRT3 protein in the spinal cord of PGC-1α KO mice compared to wt littermates (Figures 5A,C and Supplementary Figures S4A,C). In the cortex of PGC-1α KO mice *Sirt3* (Supplementary Figures 4A,C, 10) mRNA and SIRT3 protein levels also showed a decreasing trend, without reaching statistical significance (Figures 5B,D, Supplementary Figure 11), whereas *Sirt4* and *Sirt5* mRNA levels were evenly distributed (Supplementary Figures S4B,D).

**Figure 6:**
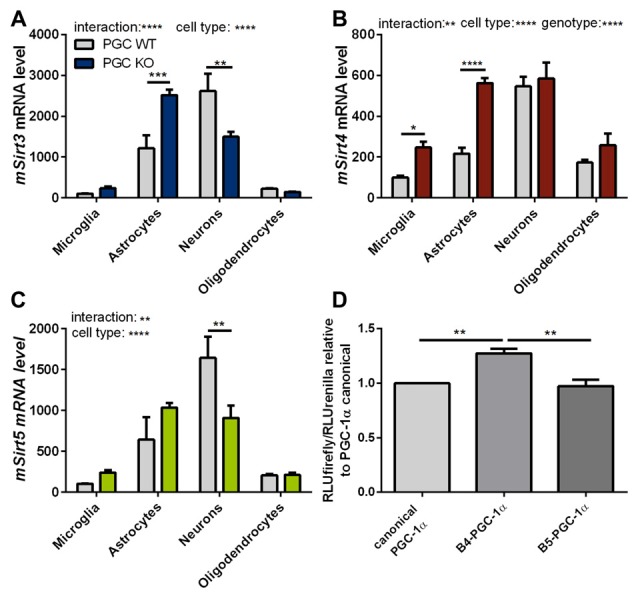
**PGC-1α isoforms influence Sirt3 mRNA levels cell type specifically.** Primary microglia, astrocytes, neurons and oligodendrocytes were cultured from B6.129-Ppargc1a^tm1Brsp^/J (PGC-1α KO) mice. *Sirt3* **(A)**, *Sirt4* **(B)** and *Sirt5* **(C)** mRNA levels were determined by qPCR and normalized to TATA box binding protein (*Tbp*), RNA polymerase 2 (*Polr2a*). The mRNA levels are shown relative to wt microglia cells. Data was analyzed by two-way ANOVA, followed by Sidak’s multiple comparison test with *n* = 4–6. The DualGlo luciferase assay (Promega) was used to determine the PGC-1α mediated activation of the Sirt3 promoter **(D)**, measured by the expression of the firefly luciferase. Neuro2a cells were co-transfected with PGC-1α isoforms (canonical PGC-1α, CNS-specific B4-PGC-1α and B5-PGC-1α; exon B4 or B5 are added at the N-terminus of canonical PGC-1α, respectively) and the renilla luciferase. Forty-eight hours after transfection the luciferase assay was performed. The activity of the firefly luciferase was normalized to the renilla luciferase activity and relative light units (RLU) are shown relative to the canonical PGC-1α levels. Data were analyzed using the Kruskal-Wallis test, followed by Dunn’s multiple comparison test with *n* = 8. Data are shown as mean ± SEM. ns = *P* > 0.05, *P ≤ 0.05, **P ≤ 0.01, ***P ≤ 0.001, ****P ≤ 0.0001.

To assess these differences at the cell-type level, we investigated the expression levels of mitochondrial sirtuins in primary neurons and different glial types isolated from PGC-1α KO mice. The distribution of *Sirt3* mRNA levels in the different cell types of PGC-1α KO mice differed significantly from wt cells. In PGC-1α KO cells the *Sirt3* mRNA levels were now highest in astrocytes, while at the same time the *Sirt3* level in neurons of PGC-1α KO mice were lower compared to wt (Figure 6A). *Sirt4* mRNA levels were higher in PGC-1α KO cells of microglia and astrocytes (Figure 6B). Examination of *Sirt5* mRNA levels showed significantly lower levels in PGC-1α KO compared to wt neurons (Figure 6C).

To investigate whether the different PGC-1α isoforms have specific effects on the transcription of *Sirt3*, we performed a luciferase reporter assay for the promoter region of *Sirt3*. Canoncial PGC-1α or representative CNS-specific PGC-1α isoforms (B4- or B5-) were overexpressed with plasmids. The luciferase reporter assays in neuro-2a cells showed a significantly higher firefly to renilla signal of the *Sirt3* promoter region in cells transfected with the CNS-specific B4-PGC-1α isoform compared to the canonical or B5-PGC-1α isoform (Figure 6D).

Our experiments showed a trend towards reduced *Sirt3* mRNA levels in PGC-1α KO mice compared to wt littermate controls. Subsequently performed luciferase activity assays pointed towards an important role for the CNS-specific B4-PGC-1α isoform in the transcriptional regulation of *Sirt3* in neurons.

### *Sirt3* and *Ppargc1a* Expression Levels in the R6/2 Mouse Model of HD

**Figure 7:**
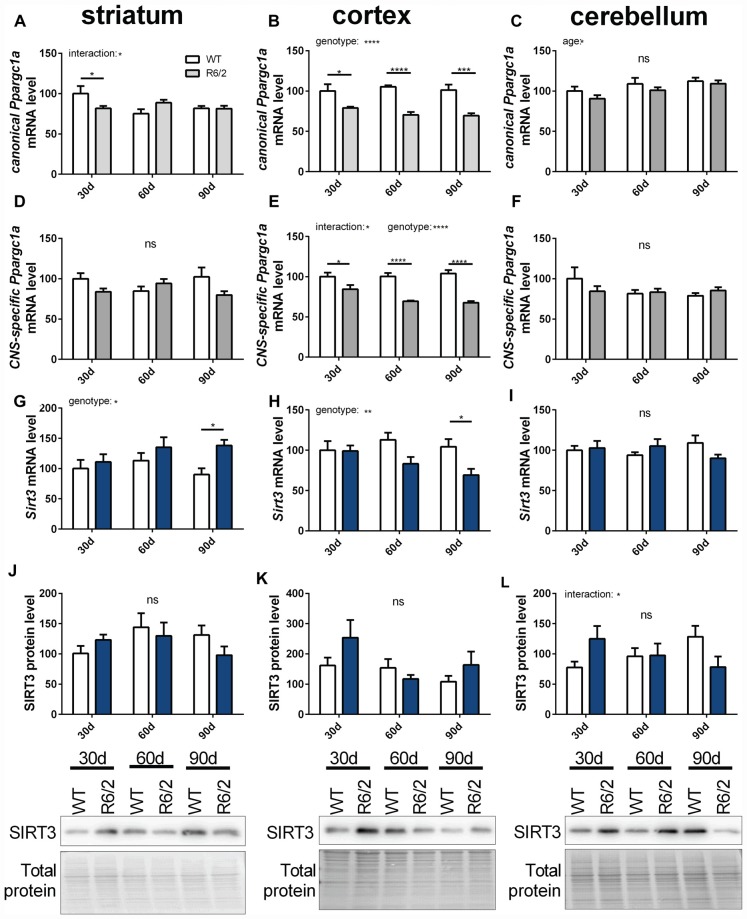
***Sirt3* mRNA levels decrease in the cortex of R6/2 mice and increase in the striatum.** mRNA levels of canonical *Ppargc1a* **(A–C)**, CNS-specific *Ppargc1a* **(D–F)** and *Sirt3* **(G–I)** were determined in the striatum **(A,D,G)**, cortex **(B,E,H)** and cerebellum **(C,F,I)** of R6/2 Huntington’s disease (HD) mice. mRNA levels were measured using qPCR and data was normalized to TATA box binding protein (*Tbp*), RNA polymerase 2 (*Polr2a*) and β-actin (*Actb*) for striatum and cortex and to *Tbp*, *Actb* and methionylaminopeptidase 1 (*Metap1*) for the cerebellum, *n* = 6–9. SIRT3 protein levels were determined for striatum **(J)**, cortex **(K)** and cerebellum **(L)** using western blot. Protein levels were normalized to total protein amount determined by MemCode staining. Data are shown relative to wt mice at 30 days of age. Data analysis was performed by two-way ANOVA followed by Sidak’s multiple comparison test with *n* = 5–6. Data are shown as mean ± SEM. ns = *P* > 0.05, *P ≤ 0.05, **P ≤ 0.01, ***P ≤ 0.001, ****P ≤ 0.0001.

In ALS and HD PGC-1α signaling is changed (Eschbach et al., 2013; Weydt et al., 2014). Therefore, we investigated the mitochondrial sirtuins and PGC-1α expression during the disease course of R6/2 mice (Mangiarini et al., 1996). Changes in mRNA levels of *canonical* and *CNS-specific* *Ppargc1a* were examined in striatum, cortex and cerebellum of R6/2 mice compared to wt controls at three strategic time points of the disease course (30, 60 and 90 days; Figures 7A–F). In the cortex both PGC-1α isoforms were significantly decreased in R6/2 mice compared to controls for all time points tested (Figures 7B,E). The two *Ppargc1a* isoforms were unchanged in the striatum and cerebellum despite of a significant reduction in the striatum of 30 days old R6/2 mice (Figures 7A,C,D,F). *Sirt3* mRNA levels decreased over the disease course in cortex tissue (Figure 7H) while in the striatum *Sirt3* mRNA levels increased (Figure 7G). In the cerebellum *Sirt3* mRNA levels remained unchanged (Figure 7I). Protein levels of SIRT3 did not change significantly over the course of disease in any of the brain regions (Figures 7J,K,L, Supplementary Figures 12–14). *Sirt4* mRNA levels were decreased in the cortex, increased in the striatum and unchanged in the cerebellum of R6/2 mice (Supplementary Table S6). *Sirt5* mRNA levels did not change in cortex and striatum and in the cerebellum the levels decreased in 90 days old mice compared to wt mice (Supplementary Table S6).

### *SIRT3* Expression Is Altered in Affected Tissue of ALS and HD Patients

**Figure 8:**
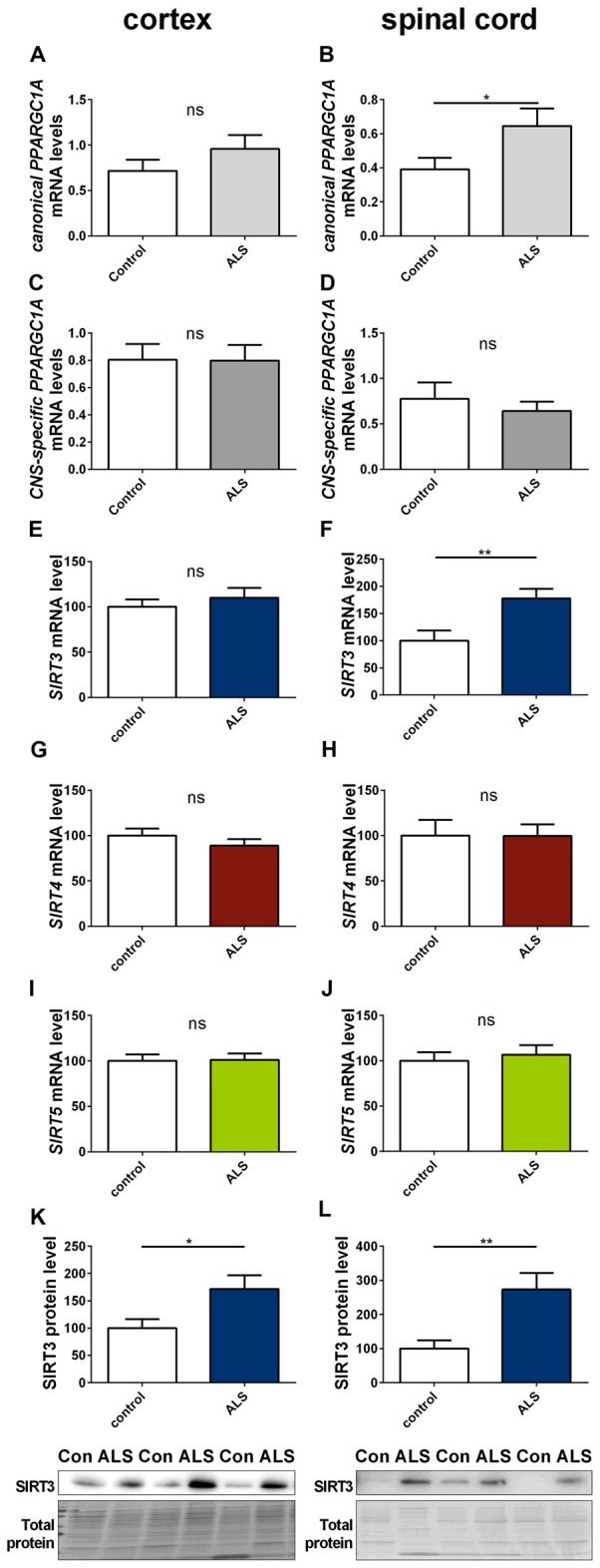
**Sirt3 levels increase in human spinal cord of amyotrophic lateral sclerosis (ALS) patients.** Human cortex **(A,C,E,G,I,K)** and spinal cord **(B,D,F,H,J,L)** were analyzed according to the canonical *PPARGC1A* **(A,B)**, CNS-specific *PPARGC1A* **(C,D)**, *SIRT3* **(E,F)**, *SIRT4* **(G,H)** and *SIRT5* **(I,J)** mRNA levels. mRNA levels were determined by qPCR and data was normalized to *TBP* and *POLR2A, n* = 10–12. SIRT3 protein levels were also determined in the human cortex **(K)** and spinal cord **(L)**. Data was normalized to total protein amount determined by MemCode staining, *n* = 12–15. Analysis was performed by the nonparametric Mann-Whitney test. Data are shown as mean ± SEM. ns = *P* > 0.05, *P ≤ 0.05, **P ≤ 0.01.

To determine to what extent our findings in animal models of neurodegenerative diseases are representative to the human conditions, we investigated the mRNA expression of *SIRT3*, canonical *PPARGC1A* and CNS-specific *PPARGC1A* in spinal cord and cortex of ALS patients and in the striatum and cerebellum of HD patients. All mRNA levels were unchanged in the cortex of ALS patients (Figures 8A,C,E), including mRNA levels of SIRT4 and SIRT5 (Figures 8G,I). For SIRT3 this was confirmed at protein level (Figure 8K). In contrast to the ALS mouse model, *SIRT3* mRNA levels were significantly increased in the spinal cord of ALS patients (Figure 8F), and this was confirmed at the protein level (Figure 8L, Supplementary Figure 16). Protein levels were also higher in the cortex of ALS patients compared to controls (Figure 8K, Supplementary Figure 15). mRNA levels of *canonical PPARGC1A* also increased in the spinal cord of ALS patients (Figure 8B). *CNS-specific PPARGC1A, SIRT4* and *SIRT5* mRNA levels were unchanged in the spinal cord of ALS patients (Figures 8D,H,J).

**Figure 9:**
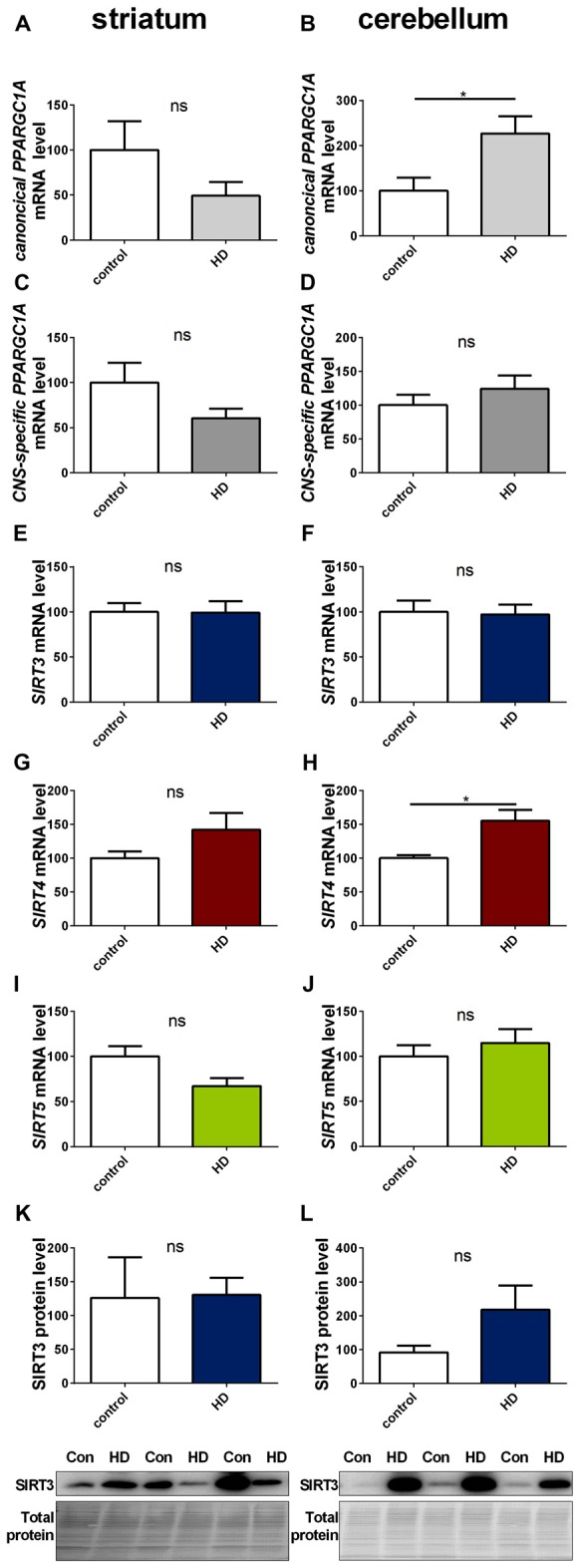
**Sirt3 levels are unchanged in human striatum and cerebellum of HD patients.** Human striatum **(A,C,E,G,I,K)** and cerebellum **(B,D,F,H,J,L)** were analyzed according to the canonical *PPARGC1A* **(A,B)**, CNS-specific *PPARGC1A* **(C,D)**, *SIRT3* **(E,F)**, *SIRT4* **(G,H)** and *SIRT5* **(I,J)** mRNA levels. mRNA levels were determined by qPCR and data was normalized to *TBP* and *POLR2A, n* = 7–10. SIRT3 protein levels were also determined in the human striatum **(K)** and cerebellum **(L)**. Data was normalized to total protein amount determined by MemCode staining, *n* = 7–9. Analysis was performed by the nonparametric Mann-Whitney test. Data are shown as mean ± SEM. ns = *P* > 0.05, *P ≤ 0.05.

We determined *SIRT3* mRNA and SIRT3 protein levels in *post mortem* striatum (Figures 9E,K, Supplementary Figure 17) and cerebellum (Figures 9F,L, Supplementary Figure 18) tissue of HD patients, revealing no significant differences. Levels of *canonical* *PPARGC1A* (Figures 9A,B) and *CNS-specific PPARGC1A* (Figures 9C,D) mRNA also did not yield any discemable patterns. The cerebella of HD patients showed increased levels of canonical *PPARGC1A* levels (Figure 9B). *SIRT4* mRNA levels were unchanged in the striatum (Figure 9G) and significantly increased in the cerebellum (Figure 9H). *SIRT5* levels were unchanged in the striatum (Figure 9I) and cerebellum (Figure 9J).

## Discussion

In this study we compared tissue specific expression levels of sirtuins in ALS and HD. We demonstrated the disease stage dependent decline of *Sirt3* mRNA expression levels and biological activity in the spinal cord of the SOD1(G93A) mouse model of ALS and in the cortex of R6/2 mice. Based on our experiments neither the neuronal cell loss in the spinal cord of the ALS mouse model nor inflammatory stimuli can fully explain the decreasing *Sirt3* mRNA levels. We suggest modulating influences of the known disease modifier PGC-1α (Eschbach et al., 2013; Weydt et al., 2014), which also co-activates the transcription of *Sirt3*. Findings on PGC-1α KO cells support a PGC-1α dependent cellular distribution of *Sirt3* mRNA levels, which possibly reflects the cell type-specific distribution of different PGC-1α isoforms. In human post mortem HD tissue and controls these findings could not be confirmed. In contrast to the mouse data of the SOD1(G93A) model *SIRT3* mRNA and protein levels increased in the spinal cord of ALS patients and remained unchanged for HD patients.

Research on tg animals is limited by the model-specific constraints and the translation of the results to humans needs to be done with caution. Despite these limitations tg animals allow for the investigation of time points and tissues that cannot be studied in human patients. We therefore put an emphasis on validating our animal findings in human tissues wherever possible.

### *Sirt3* Signals *In Vitro* vs. *In Vivo*

Highest levels of *Sirt3* mRNA were expressed in the spinal cord and brain stem, two CNS regions affected in ALS (Kaur et al., 2016). Specifically decreased *Sirt3* mRNA levels were measured in spinal cord and brain stem of the ALS SOD1(G93A) mouse model during the course of disease, indicating a disease specific reduction. The stability of *Sirt4* and *Sirt5* mRNA levels, two residual mitochondrial sirtuins, combined with the unchanged sirtuin levels in the control region hippocampus further supported the selective decline of *Sirt3* in spinal cord and brain stem.

The decline of *Sirt3* levels in SOD1(G93A) mice during the course of disease corresponded to the canonical and CNS-specific levels of *Ppargc1a*, which were published previously (Bayer et al., 2017). This is not surprising considering the fact that PGC-1α regulates *Sirt3* at the transcriptional level (Kong et al., 2010; Giralt et al., 2011).

SIRT3 protein levels showed a disease stage dependent decline in the spinal cord of SOD1(G93A) tg mice, reaching the same levels as wt mice at 130 days of age. SIRT3 levels in the wt animals did not change. A decline in *Sirt3* mRNA and protein levels as well as in SIRT3 function is in line with findings described in aging rats (Zeng et al., 2014), suggesting features of accelerated aging regarding SIRT3 in the SOD1(G93A) model. It remains to be determined why SIRT3 levels are higher in SOD1(G93A) tg mice at the age of 60 days compared to wt littermate controls. It is well established that cellular and molecular precede the onset of overt symptoms in this model (Dal Canto and Gurney, 1995). Furthermore, the analysis of the acetylation status of the mitochondrial SIRT3 target SOD2 (Tao et al., 2010), showed increasing acetylation levels over the course of disease, when normalized to the total amount of SOD2. This argues for a decreased activity of SIRT3, which is in line with the work of Song et al. (2013), showing a decline in the sirtuin activity of spinal cord tissue lysates.

Narrowing our findings down from the tissue level to the cellular level, we detected highest mRNA and protein levels of mitochondrial sirtuins in primary neuron cultures, followed by astrocytes. Given the high SIRT3 levels in neurons, this cell type appears to determine the overall brain tissue levels. But this scenario would also predict decreasing levels of *Sirt4* and *Sirt5*, which however show a stable distribution in the spinal cord during the course of disease. As all mitochondrial sirtuins are highly expressed in neurons, the selective decrease in SIRT3 is likely not a simple reflection of neuronal loss. Our results did not correlate well with the mRNA expression levels in the Stanford Brain RNAseq database^2^ (Zhang et al., 2014). In this database mRNA levels of all mitochondrial sirtuins are quite homogenous among neurons, astrocytes and oligodendrocytes (in the database referred to as “OPC”). Discrepancies between our data and the published database might reflect procedural differences, e.g., in cell selection or the culturing process.

A further methodological limitation of both approaches is that they rely on neonatal or embryonic tissue respectively, which are obtained well before disease onset in our disease models. Therefore, developmental differences likely limit the applicability of these data to adult *in vivo* results. When comparing cell culture data, one has to additionally take into account that the prevalence of the different cell types changes during tissue inflammation (Lull and Block, 2010). These changes in the cell type composition inevitably confound tissue levels of mitochondrial sirtuins in ALS.

Focusing on inflammation in the affected tissues of ALS (Philips and Robberecht, 2011), the involvement of SIRT3 in immune cell activation in the CNS was probed. The role of SIRT3 in inflammatory processes had been shown previously in peripheral blood monocytes (Liu et al., 2015; Traba et al., 2015). In LPS-stimulated primary microglia *Sirt3*, *Sirt4* and *Sirt5* mRNA levels decreased initially, but recovered after 24 h. In primary astrocytes sirtuin mRNA levels were unchanged after LPS stimulation.

The decreasing levels of *Sirt3* in primary microglia cells upon LPS stimulation could explain the declining *Sirt3* mRNA levels in the spinal cord of SOD1(G93A) animals over the disease course. It is unlikely that this LPS induced reduction explains the total *Sirt3* tissue levels in the spinal cord of the SOD1(G93A) mice because *Sirt4* and *Sirt5* mRNA levels were decreased in the LPS-stimulated primary microglia cells but not in the tissue samples. We therefore conclude that either the contribution in the changes in mitochondrial sirtuin levels of microglia cells is negligible in the evaluation of tissue levels or the method of primary microglia cells derived from newborn pups is not appropriate to model adult onset changes.

### PGC-1α in ALS and HD

Based on the similarities in hypermetabolism (Djousse et al., 2002; Dupuis et al., 2011) and mitochondrial dysfunction (Lin and Beal, 2006) between ALS and HD patients, we aimed to compare both diseases with regard to PGC-1α and SIRT3 expression. The metabolic master regulator PGC-1α is a disease modifier in ALS and HD (Eschbach et al., 2013; Weydt et al., 2014) and also controls the expression of *Sirt3*.

To probe their mutual relationship, we determined whether the absence of PGC-1α influences the expression levels of *Sirt3*. The functional PGC-1α KO mouse model, which lacks exons 3–5 of the PGC-1 α gene (Lin et al., 2004) indeed showed a tendency towards decreased levels of *Sirt3* mRNA levels and SIRT3 protein levels in the spinal cord and cortex. The deleted region is highly conserved across species and includes a motif mediating the interaction with many nuclear receptors (Lin et al., 2004). A reduction in *Sirt3* mRNA levels in PGC-1α KO mice was shown previously (Kong et al., 2010; Giralt et al., 2011). The changes we detected did not reach significance, and larger group sizes are needed to reach conclusive results.

Our experiments on primary cultured cells demonstrated a cell type specific redistribution of mitochondrial sirtuins in PGC-1α KO cells, with highest *Sirt3* mRNA levels in astrocytes instead of neurons. The cell type specific expression of canonical and CNS-specific PGC-1α isoforms provides a possible explanation for the discrepant *Sirt3* mRNA levels in different brain derived cell types of PGC-1α KO cells.

PGC-1α regulates different mitochondrial pathways in diverse tissues and cell types via a cell type and tissue specific expression of splice variants and isoforms (Martínez-Redondo et al., 2015). Several PGC-1α isoforms are expressed under a brain-specific promoter, restricting the expression to the CNS (Soyal et al., 2012). Experiments in our lab showed restricted expression of CNS-specific isoforms (exon B1–B4) in neurons, while the canonical isoform is also expressed in glia cells (Bayer et al., 2017). Of note, these different isoforms have different transcriptional co-activator activities (Bayer et al., 2017).

The roles of PGC-1α are very diverse and its functions are numerous. In the present study the experiments were focused on CNS tissues. We used a luciferase reporter assay to test whether different PGC-1α isoforms regulate *Sirt3* transcription differentially. The B4-PGC-1α construct activated the *Sirt3* promoter stronger than the B5-PGC-1α or the canonical PGC-1α constructs. These findings suggest that the higher neuronal *Sirt3* mRNA levels reflect a neuron-specific expression of the CNS-specific PGC-1α isoforms. Further experiments are necessary to characterize the cell type specific PGC-1α isoform, which is mainly responsible for *Sirt3* transcription. While our experiments speak to the cell type specific distribution of *Sirt3* mRNA levels in wt cells, they do not fully explain why *Sirt3* mRNA levels increase drastically in astrocytes upon the lack of exons 3–5 in PGC-1α. One limitation of the luciferase reporter assay is the use of neuro-2a cells instead of primary cultured neurons, which mimick the murine brain more closely. But primary cultured neurons are profoundly influenced by confounding factors such as harvesting and culturing, developmental differences and the absence of interactions with other cell types. Thus these results need to be interpreted with caution.

### Differences between ALS and HD

PGC-1α and SIRT3 levels were compared in the R6/2 model of HD and the SOD1(G93A) model of ALS. *Canonical* and *CNS-specific Ppargc1a* mRNA levels were lower in the cortex of R6/2 mice compared to wt littermate controls. These results confirmed previous publications reporting reduced levels of the *canonical* *Ppargc1a* isoform on mRNA level in the cortex (Hering et al., 2015). Reduced levels were also reported for the striatum of HD mice (Cui et al., 2006; Weydt et al., 2006; Hering et al., 2015). In our hands unchanged levels of the different *Ppargc1a* isoforms were detected in the striatum of R6/2 mice. In the NLS-N171-82Q tg HD mouse model reduced mRNA and protein levels were reported (Chaturvedi et al., 2010). Lower *Sirt3* mRNA levels were detected in the cortex of 90 days old R6/2 HD mice, paralleling the changes in *Ppargc1a* mRNA levels. *Sirt3* mRNA levels were higher in the striatum of 90 days old R6/2 mice. The cerebellum was examined as a relatively spared brain area in HD and no significant changes were found, demonstrating brain region specific changes in *Ppargc1a* and *Sirt3* mRNA levels. Protein levels of SIRT3 did not result in any significant changes in the brain regions examined.

In contrast to the cortex, the striatum of 90 days old R6/2 mice showed higher levels of *Sirt3* mRNA. This differs from the decreased levels of *Sirt3* in the spinal cord and brain stem of ALS SOD1 (G93A) mice. Although PGC-1α is involved in the pathogenesis of both diseases, intriguing remain. For instance the effect of a SNP, localized close to the CNS-specific *Ppargc1a* promoter, is protective in HD (Soyal et al., 2012) while in ALS it is clearly detrimental (Eschbach et al., 2013). Different PGC-1α involving pathomechanisms could dysregulate *Sirt3* in a different manner in ALS and HD. Also, in HD transcriptional defects manifest much earlier in the disease course than in ALS. More than 200 mRNAs were described to be dysregulated in HD brains (Hodges et al., 2006; Anderson et al., 2008; Becanovic et al., 2010). Mutant huntingtin interacts with transcription factors, co-transcriptional activating factors such as PGC-1α and histone acetyl transferases (Li and Li, 2004; Anderson et al., 2008; Johri et al., 2013). While in SOD1 related ALS mRNA metabolism is not prominently affected, other ALS causing mutations do involve compromised transcription (Walsh et al., 2015).

### Translational Aspects

Analysis of human cortical tissue of ALS patients did not show any changes in mitochondrial sirtuin levels. *Canonical PPARGC1A* and *SIRT3* mRNA levels as well as SIRT3 protein levels were higher in human post mortem ALS spinal cord tissue, while *CNS-specific PPARGC1A* mRNA levels were unaltered. This constellation argues against the simplistic hypothesis that *CNS-specific PPARGC1A* mRNA levels alone determine *SIRT3* mRNA levels. Furthermore, these findings are not in line with the decreased *Sirt3* levels in the SOD1(G93A) spinal cords.

We examined *SIRT3, 4, 5, canonical and CNS-specific PPARGC1A* mRNA and SIRT3 protein levels in post mortem striatum and cerebellum from HD patients. Mainly the results revealed no differences between patient and control tissues, despite higher levels of *canonical PPARCG1A* and *SIRT4* mRNA. SIRT3 protein levels showed a trend towards an increase compared to controls. The unchanged levels or *CNS-specific Ppargc1a* and *Sirt3* in human HD patient tissue indicates the lack of significance of this pathway for HD pathogenesis. The R6/2 HD mouse model showed significantly higher *Sirt3* mRNA levels and unchanged SIRT3 protein levels in the striatum. There are many non-mutually exclusive explanations for these discrepancies. They could be due to the species-specific degree of neuronal cell loss in the striatum. Also tg models have much larger repeat expansions compared to patients. In advanced human HD stages a striatal neuron loss of up to 90% is found (Vonsattel et al., 1985) compared to 12% in 90 days old R6/2 mice (Dodds et al., 2014). Brains from R6/2 mice show a 19% reduction in size (Mangiarini et al., 1996). R6/2 mice do not show the specific progressive atrophy of the caudate, putamen and globus pallidus associated with astrogliosis (Mangiarini et al., 1996), which is striking for human brains of higher HD grade (Vonsattel et al., 1985; Myers et al., 1991).

To overcome the limitations of exon-1 fragment models such as the R6/2 line, we turned to a genetically more accurate model, the knock-in HD mouse model, which expresses the full length huntingtin gene with a normal (Q20) or an expanded (Q175) CAG repeat tract (Langfelder et al., 2016). The transcriptomic RNAseq analysis of the knock-in mouse model showed a significant increase of *Sirt3* mRNA levels and a significant decrease of *Ppargc1a* levels in the striatum of 6 and 10 months old Q175 mice compared to Q20 control mice (Langfelder et al., 2016). mRNA levels in the cortex did not change in this mouse model. The knock-in mouse is considered to be genetically more accurate than R6/2. The significant decrease in *Ppargc1a* mRNA levels of the knock-in mouse model in the striatum is reminiscent of the trend towards a decrease that we observed in the human tissue.

In summary, we provided evidence that *Sirt3* levels and function *decrease* during the course of disease in affected tissues of the SOD1(G93A) ALS mouse model, whereas *Sirt3* mRNA levels *increased* in the striatum of the R6/2 mouse model. Furthermore, inflammatory processes and neuronal loss possibly contribute to the decrease of *Sirt3* mRNA levels during the course of disease in ALS SOD(G93A) animals. However, additional mechanisms have to be involved, as the other mitochondrial sirtuins remain stable. Moreover, we could show the cell type-specific expression pattern of *Sirt3*, which is highest in neurons, is probably predominantly regulated by CNS-specific Ppargc1a isoforms. *Sirt3* levels in affected tissues of HD mice differed from ALS mice, confirming PGC-1α as a common modulator of disease specific signaling pathways. Further studies are necessary to fully elucidate the involvement of SIRT3 and PGC-1α in the pathology of ALS and HD.

## Ethics Statement

In our study we used human post mortem brain tissue. This study was carried out in accordance with the recommendations of the local brain bank at Ulm University with written informed consent from all subjects. All subjects gave written informed consent in accordance with the Declaration of Helsinki. This study was carried out in accordance with the recommendations of the Animal Ethics committee of the Regional Steering Committee Tübingen. The protocol was approved by the Regional Steering Committee Tübingen and the local animal facility.

## Author Contributions

EB and AW were responsible for the concept and design of the work. Data acquisition was performed by EB, HB, JH and NP. Data analysis and interpretation was performed by EB and HB. ACL provided clinical data of the ALS autopsy cases. KSL, PW and AW critically revised the manuscript. All authors reviewed and approved the manuscript.

## Conflict of Interest Statement

The authors declare that the research was conducted in the absence of any commercial or financial relationships that could be construed as a potential conflict of interest.

## Footnotes

^1^https://www.jax.org/strain/002726 and https://www.jax.org/strain/008597

^2^http://web.stanford.edu/group/barres_lab/cgi-bin/igv_cgi_2.py?lname=sirt3

## Abbreviations

ALS: amyotrophic lateral sclerosis
B2M: beta-2 microglobulin
BCA: bicinchoninic acid
CNS: central nervous system
CS: citrate synthase
Dloop: displacement loop
DMEM: dulbecco’s modified eagle medium
FBS: fetal bovine serum
FUS: FUS RNA binding protein
HD: Huntington’s disease
HRP: horseradish peroxidase
KO: knock-out
LPS: lipopolysaccharide
mtDNA: mitochondrial DNA
PGC-1α: peroxisome proliferative-activated receptor gamma coactivator 1 alpha
PVDF: polyvinylidene fluoride
qPCR: quantitative real-time PCR
ROS: reactive oxygen species
RLU: relative light units
Sirt3: sirtuin 3
Sirt4: sirtuin 4
Sirt5: sirtuin 5
SNP: single nucleotide polymorphism
SOD1: superoxide dismutase 1
SOD2: superoxide dismutase 2
tg: transgenic
wt: wild type.
