# Supplementary material for: Comparison of Sirtuin 3 Levels in ALS and Huntington’s Disease—Differential Effects in Human Tissue Samples vs. Transgenic Mouse Models
Source: Front Mol Neurosci. 2017 May 26;10:156. doi: 10.3389/fnmol.2017.00156 (PMC5445120; doi:10.3389/fnmol.2017.00156)
Supplement: Supplementary file 1 [file Data_Sheet_1.pdf]

Supplementary Material

Comparison of sirtuin 3 levels in ALS and Huntington's disease – differential effects in human tissue samples vs. transgenic mouse models

Eva Buck, Hanna Bayer, Katrin S. Lindenberg, Johannes Hanselmann, Noemi Pasquarelli, Albert C. Ludolph, Patrick Weydt*, Anke Witting

*** Correspondence:**
Patrick Weydt
patrick.weydt@ukbonn.de

1 Supplementary Figures and Tables

1.1 Supplementary Figures

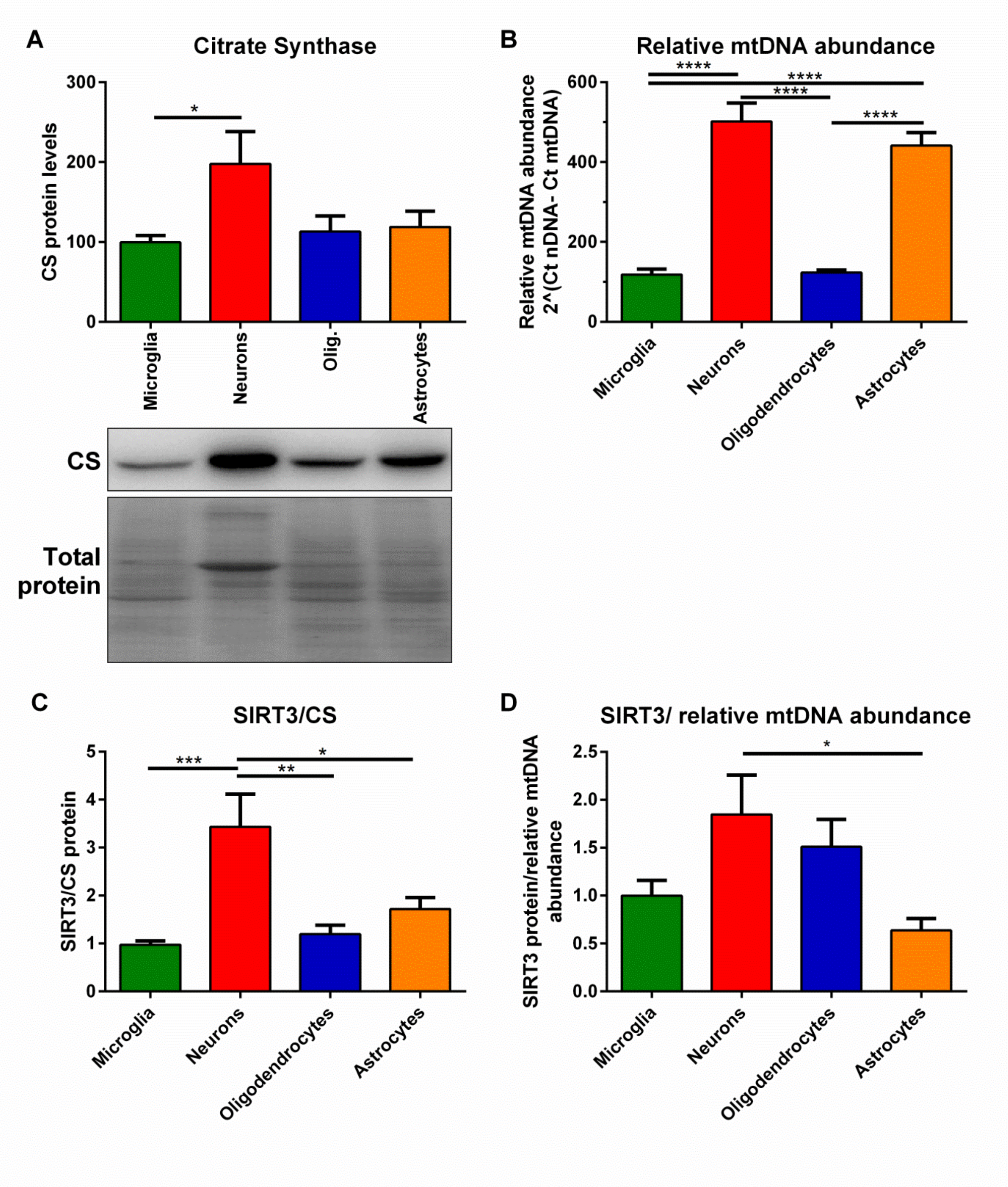

Supplementary Figure 1. Mitochondrial mass is highest in neurons. Mitochondrial mass was determined in primary cultured microglia, neurons, oligodendrocytes and astrocytes of C57BL/6J mice using citrate synthase (CS) protein levels (**A**) and the relative mitochondrial DNA (mtDNA) abundance (**B**). Ratios of SIRT3 protein levels to either the CS levels (**C**) or the mtDNA copy number (**D**) were determined. Data were analyzed by the one-way ANOVA followed by Tukey's

multiple comparison test, n = 5 – 8. Data are shown as mean ± SEM. ns=P>0.05, *=P≤0.05, **=P≤0.01, ***=P≤0.001, ****=P≤0.0001.

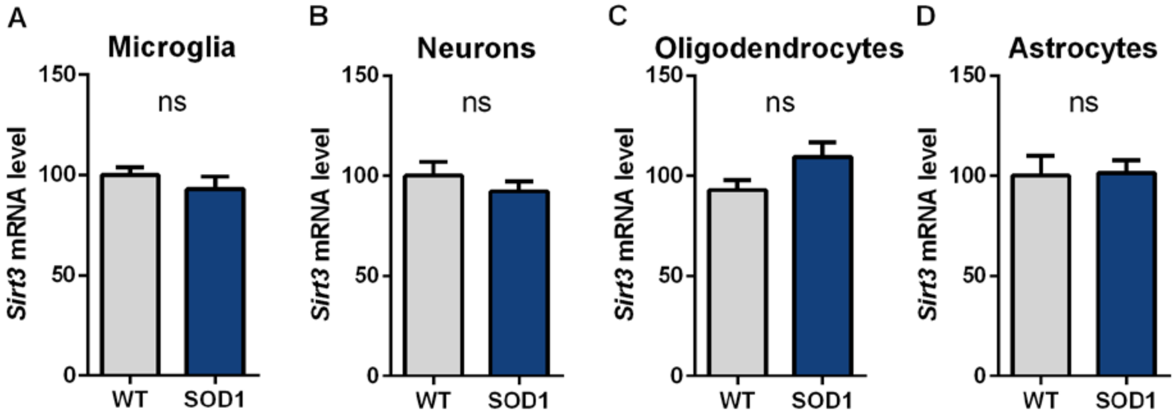

Supplementary Figure 2. *Sirt3* mRNA levels are unchanged comparing SOD1(G93A) derived primary cells to controls. *Sirt3* mRNA levels were determined in microglia (**A**), neurons (**B**), oligodendrocytes (**C**) and astrocytes (**D**) of B6SJL-Tg(SOD1*G93A)1Gur/J (SOD(G93A)) mice. qPCR experiments were performed and data was normalized to TATA box binding protein (*Tbp*), RNA polymerase 2 (*Polr2a*) (for **B**, **C**) and additionally to β-actin (*Actb*) (for **A**, **D**) (neurons n = 5, microglia n = 25, oligodendrocytes n = 6, astrocytes n = 9). Data were analyzed by unpaired student's t-test and results are shown as mean ± SEM.

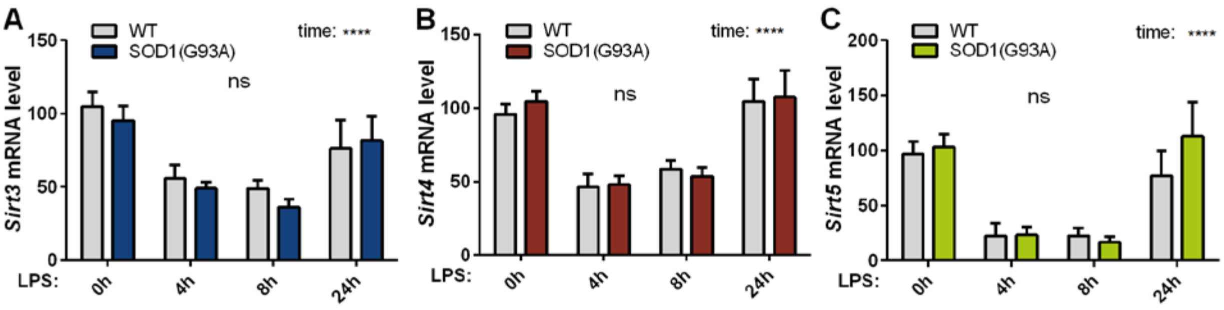

Supplementary Figure 3. Primary microglia of SOD1(G93A) mice show no differences in mitochondrial sirtuin levels upon LPS stimulation. Cultured primary microglia cells of B6SJL-Tg(SOD1*G93A)1Gur/J (SOD(G93A)) mice were treated with 1 µg/ml LPS for 0, 4, 8 and 24 h. mRNA levels of *Sirt3* (**A**), *Sirt4* (**B**) and *Sirt5* (**C**) were determined by qPCR and normalized to TATA box binding protein (*Tbp*), RNA polymerase 2 (*Polr2a*). Data are shown relative to the untreated wild type control. Analysis was performed using two-way ANOVA followed by Sidak's multiple comparison test with n = 3 – 5. Data are shown as mean ± SEM. Significance is indicated by ****=P≤0.0001.

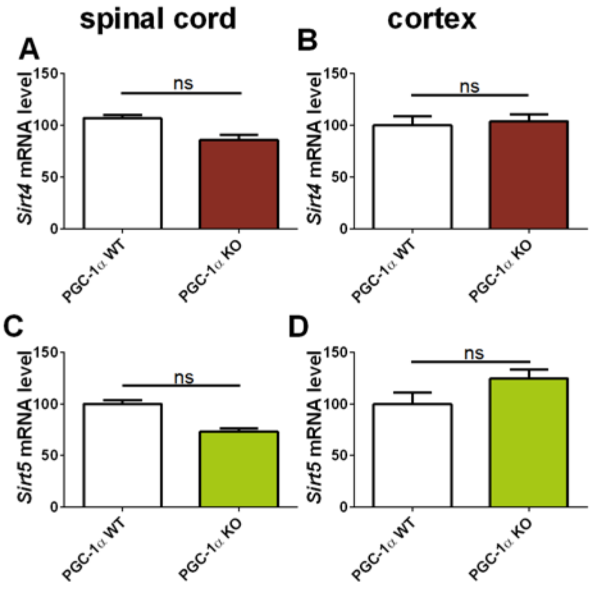

Supplementary Figure 4. *Sirt3* levels show a decreasing tendency in the spinal cord and cortex of PGC-1α KO mice. *Sirt4* (**A**, **B**) and *Sirt5* (**C, D**) mRNA levels were determined by qPCR in the spinal cord (**A**, **C**) and cortex (**B**, **D**) of 90 d old B6.129-Ppargc1a^{tm1Brsp}/J (PGC-1α KO) mice and respective controls. mRNA levels were normalized to TATA box binding protein (*Tbp*), RNA polymerase 2 (*Polr2a*) and β-actin (*Actb*). Levels are shown in percent of the wild type control. The nonparametric Mann-Whitney test was used for analysis with n = 3. Data are shown as mean ± SEM.

1.2 Additional Figures providing raw pictures of Western Blots

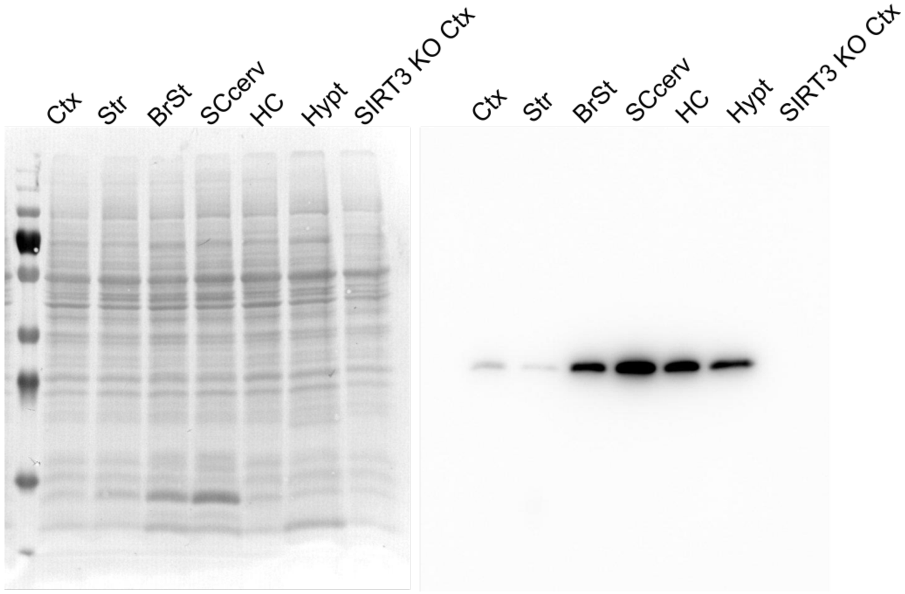

Supplementary Figure 5. Membranes Figure 1D. On the left side the protein stain is indicated and on the right side the SIRT3 protein membrane is shown.

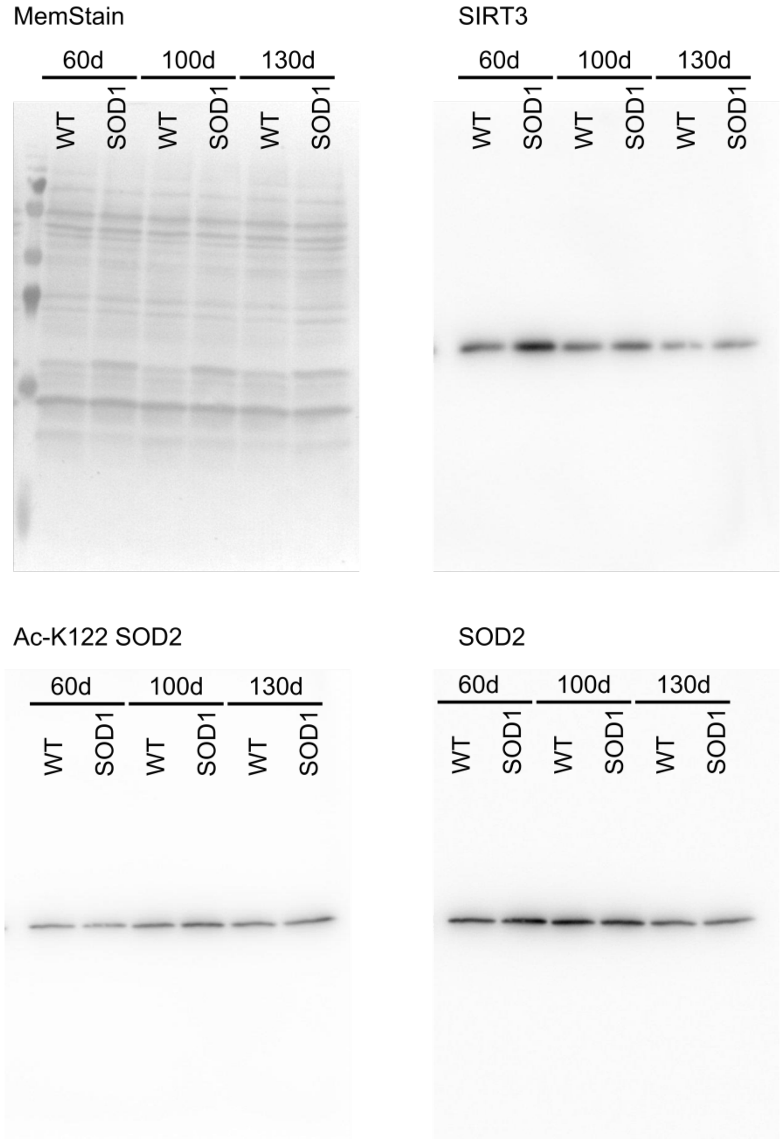

Supplementary Figure 6. Membranes Figure 2F. Membranes for Figure 2F are shown including the total protein on the MemStain, SIRT3, Ac-K122 SOD2 and SOD2 protein bands.

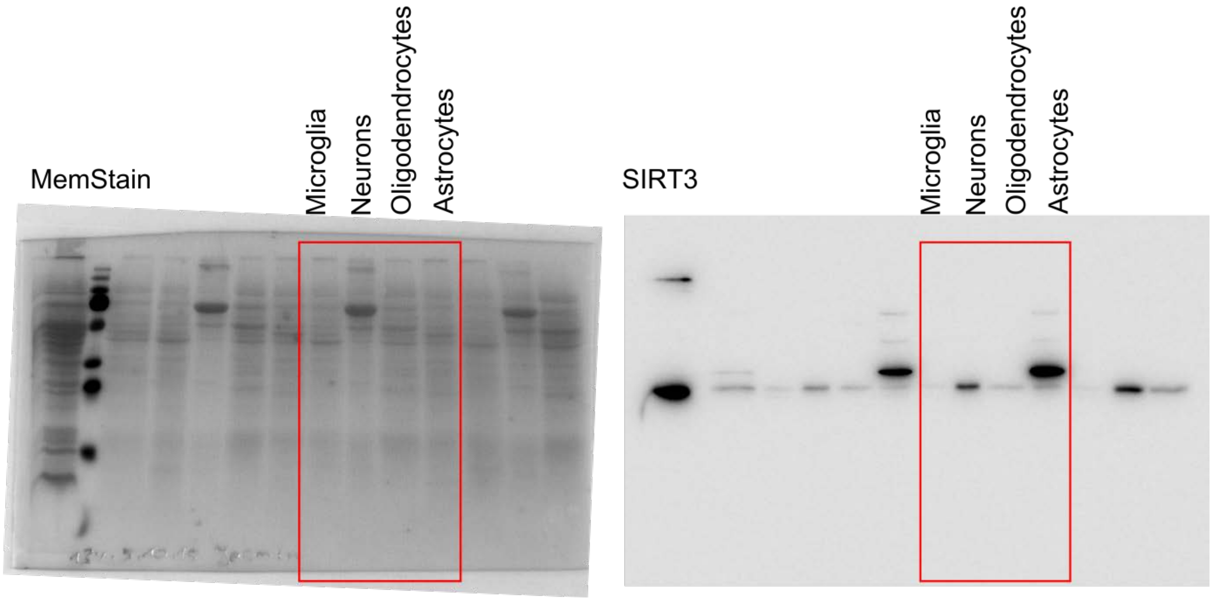

Supplementary Figure 7. Membranes Figure 3D. Membranes showing the total protein amount stained by MemStain (left) and the SIRT3 protein (right). The bands cut for the Figure are marked red. As indicated in Figure 3D there is one prominent additional band for astrocytes.

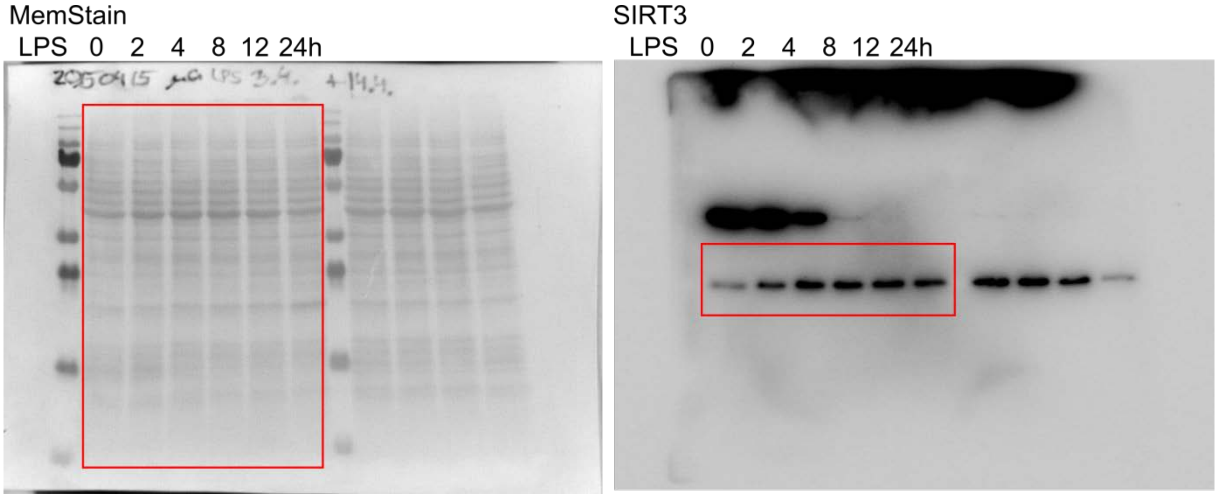

Supplementary Figure 8. Membranes Figure 4G. Microglia cells treated with 1µg/ml LPS for different time points. Total protein amount is shown left. SIRT3 protein is shown on the right. The bands used for the figure are highlighted red.

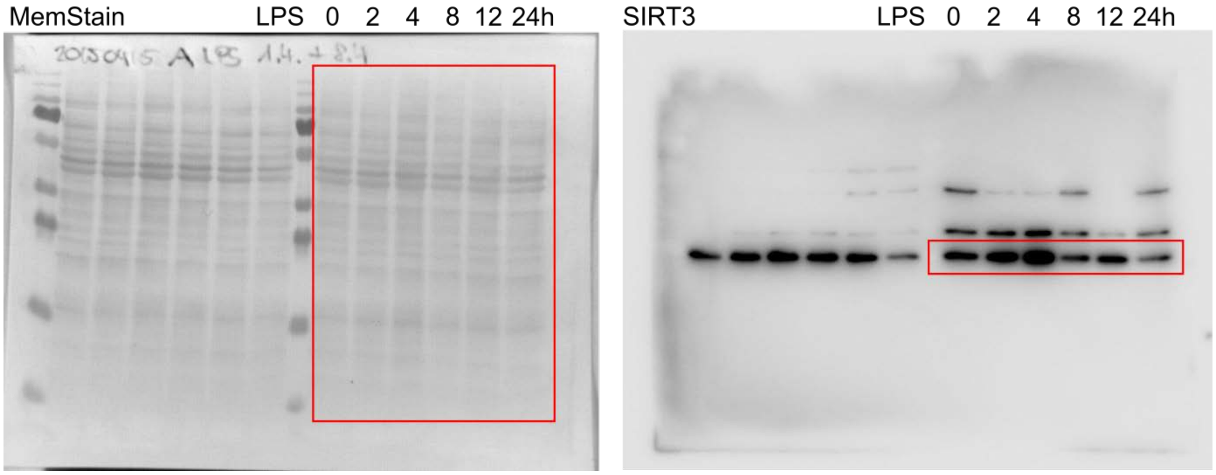

Supplementary Figure 9. Membranes Figure 4H. Astrocytes treated with 1µg/ml LPS for different time points. Total protein amount is shown left. SIRT3 protein is shown on the right. The bands used for the figure are highlighted red.

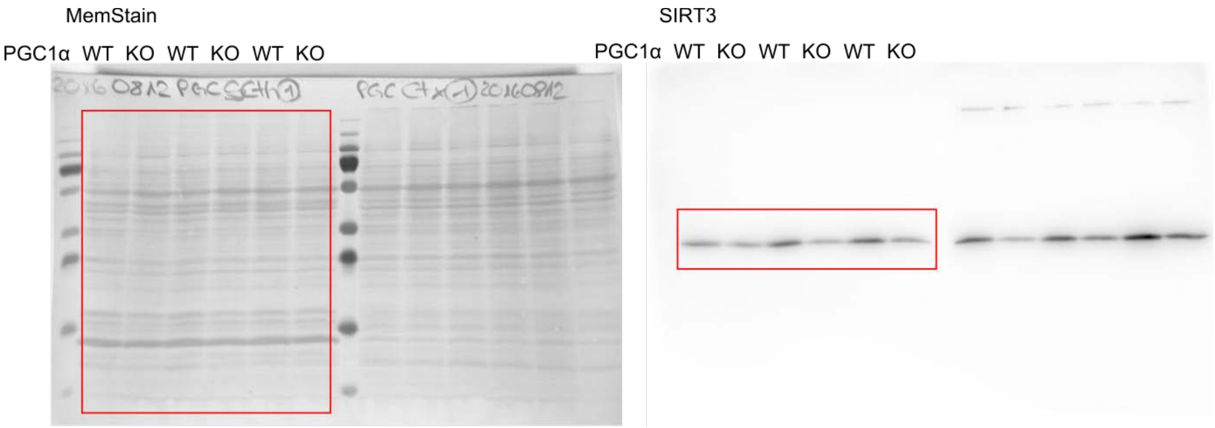

Supplementary Figure 10. Membranes Figure 5C. Spinal cord of PGC-1α KO mice compared to wt mice. Total protein amount is shown left. SIRT3 protein is shown on the right side. The bands used for Figure 5G are highlighted in red.

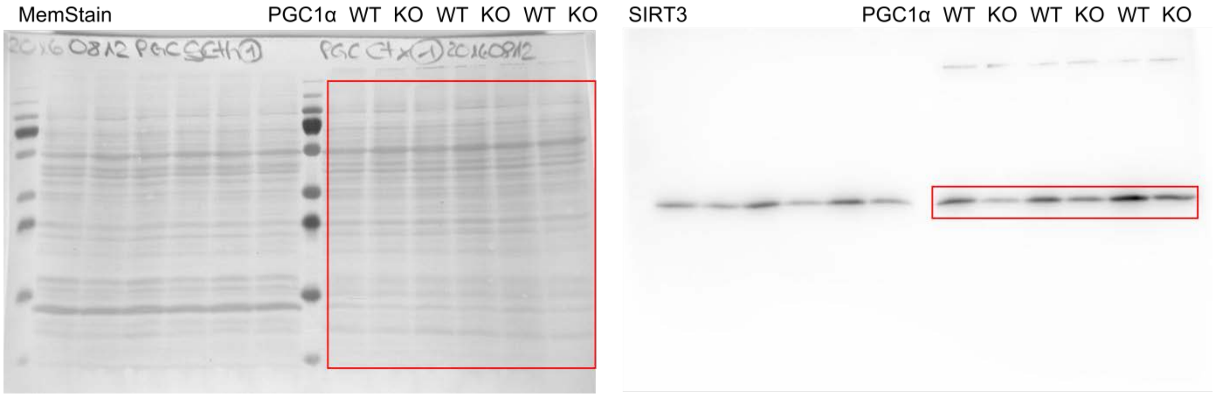

Supplementary Figure 11. Membranes Figure 5D. Cortex of PGC-1α KO mice compared to wt mice. Total protein amount is shown left. SIRT3 protein is shown on the right side. The bands used for Figure 5H are highlighted in red.

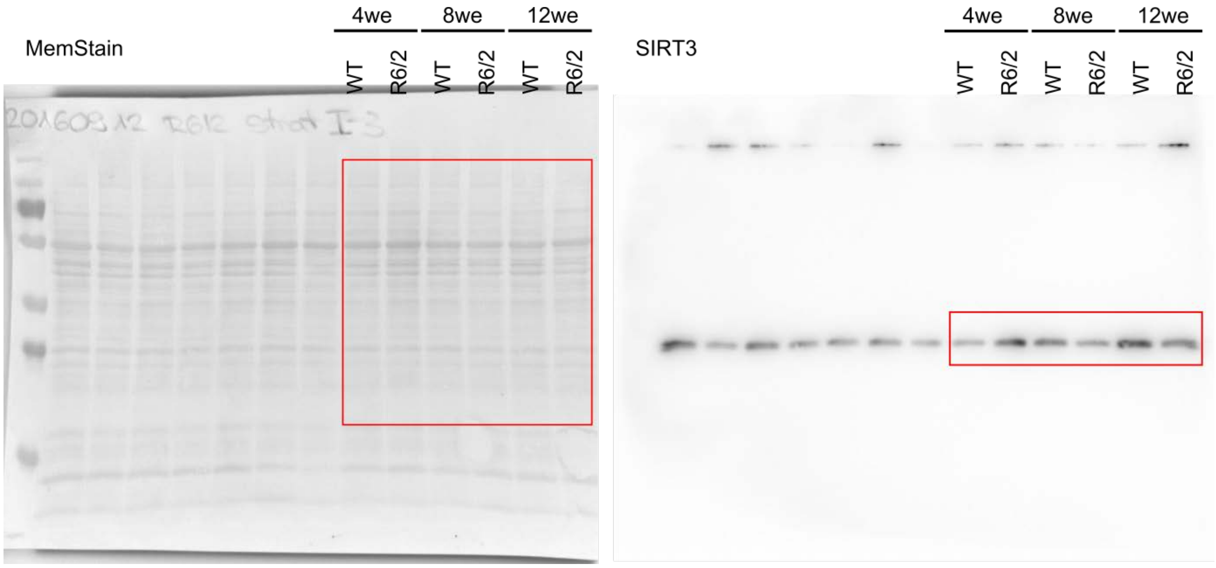

Supplementary Figure 12. Membranes Figure 7J. Striatum of R6/2 mice comparing wt and tg animals at the age of 30, 60 and 90 days. Total protein levels determined by MemStain are shown left and the SIRT3 protein levels are shown at the right. Bands used for the respective figure are labeled in red. Two different sets of the experiment are loaded on one membrane separated by one inter-membrane calibration sample, which was the same on all membranes. Therefore 13 samples instead of 12 are loaded.

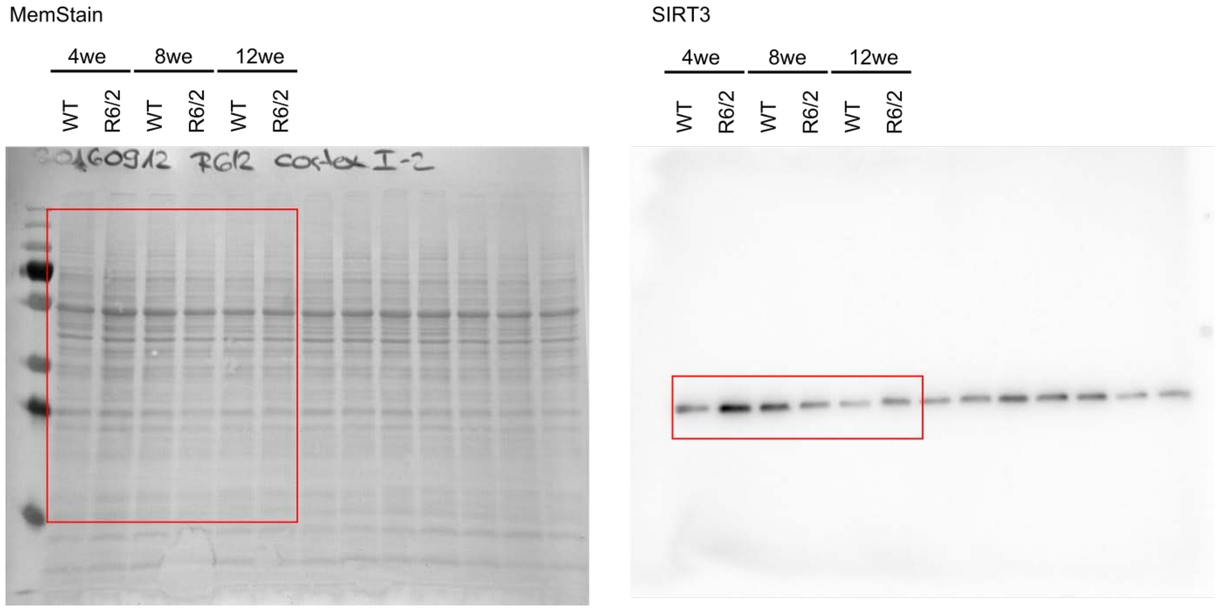

Supplementary Figure 13. Membranes Figure 7K. Cortex of R6/2 mice comparing wt and tg animals at the age of 30, 60 and 90 days. Total protein levels determined by MemStain are shown left and the SIRT3 protein levels are shown at the right. Bands used for the respective figure are labeled in red.

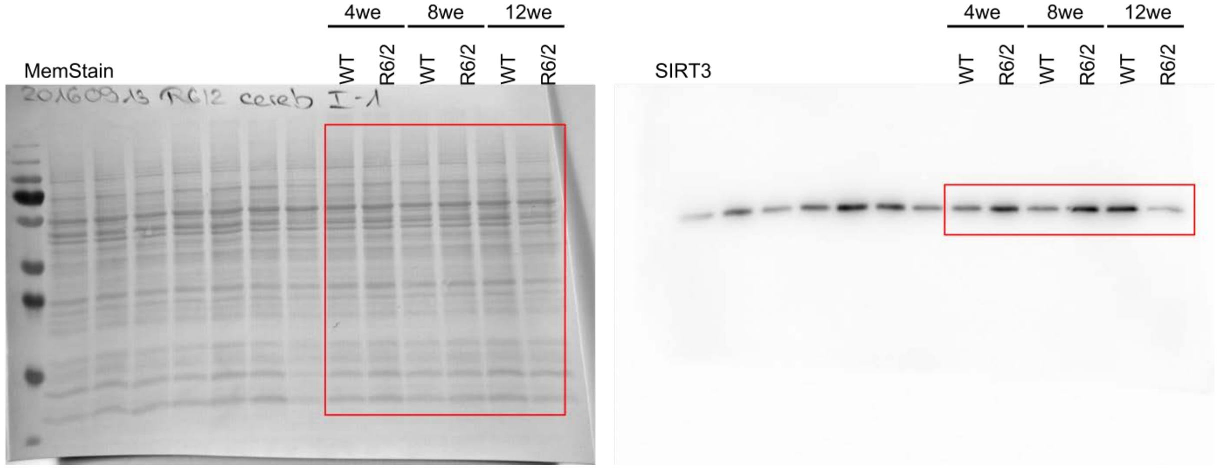

Supplementary Figure 14. Membranes Figure 7L. Cerebellum of R6/2 mice comparing wt and tg animals at the age of 30, 60 and 90 days. Total protein levels determined by MemStain are shown left and the SIRT3 protein levels are shown at the right. Bands used for the respective figure are labeled in red.

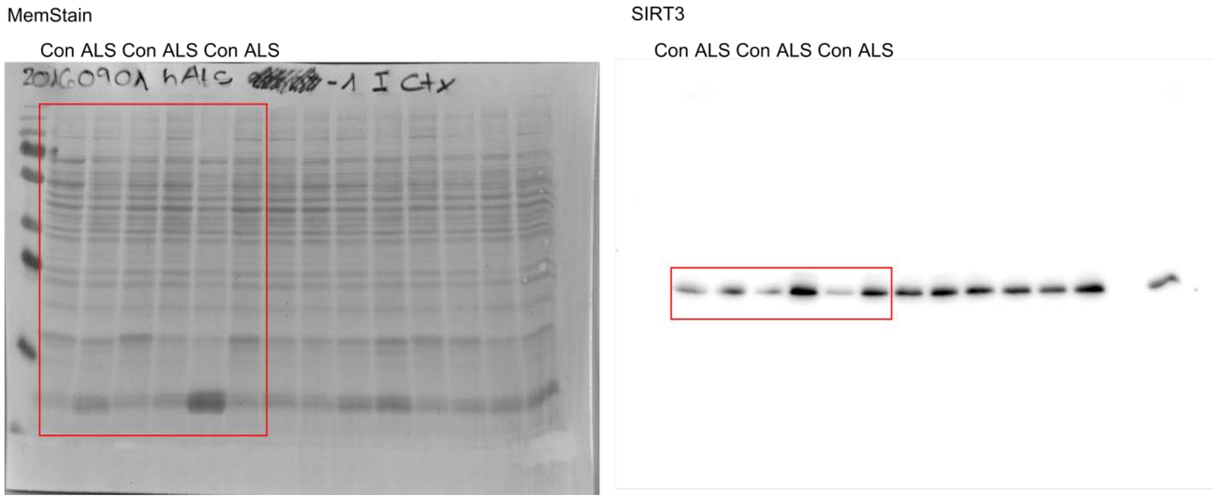

Supplementary Figure 15. Membranes Figure 8K. Human cortex of ALS patients and controls are shown. On the left side the total protein amount is determined by MemStain. The right side shows the SIRT3 membrane. Bands used for the respective figure are labeled in red.

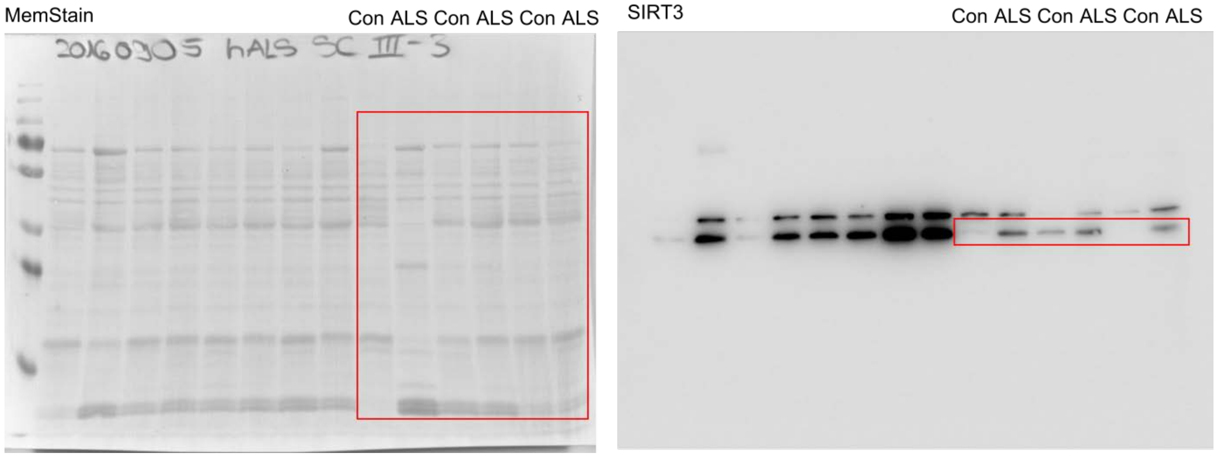

Supplementary Figure 16. Membranes Figure 8L. Human spinal cord of ALS patients and controls are shown. On the left side the total protein amount is determined by MemStain. The right side shows the SIRT3 membrane. Bands used for the respective figure are labeled in red.

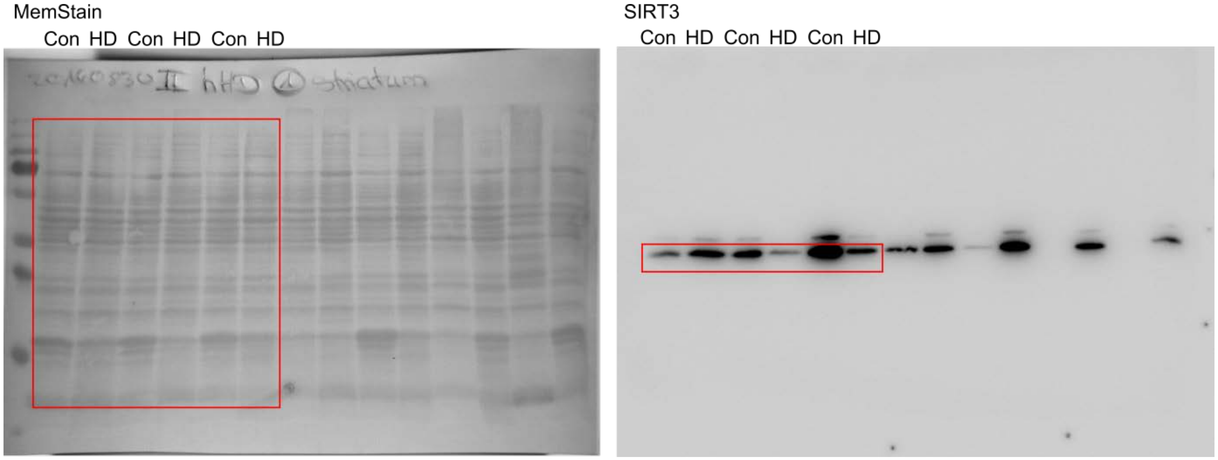

Supplementary Figure 17. Membranes Figure 9K. Human striatum of HD patients and controls are shown. On the left side the total protein amount is determined by MemStain. The right side shows the SIRT3 membrane. Bands used for the respective figure are labeled in red.

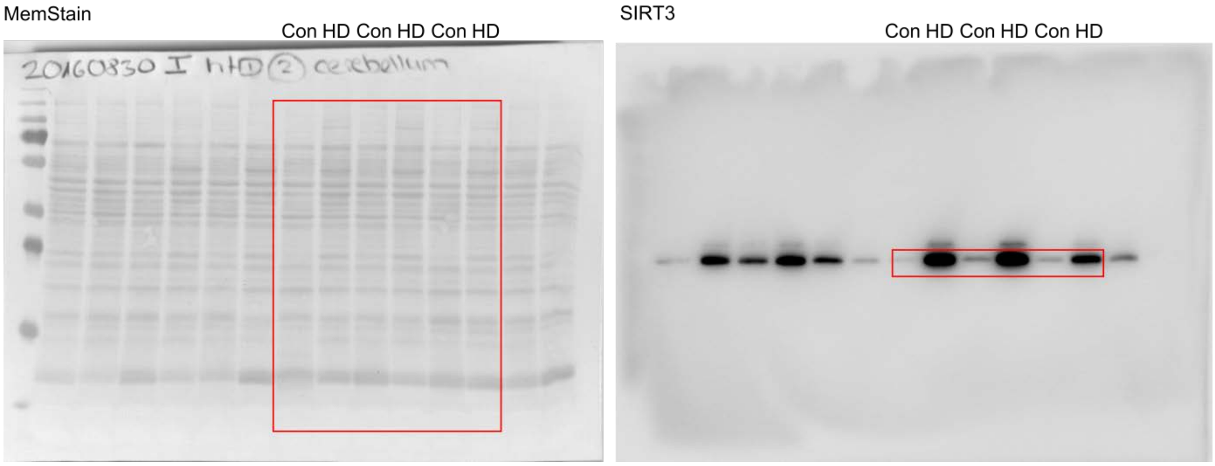

Supplementary Figure 18. Membranes Figure 9L Human cerebellum of HD patients and controls are shown. On the left side the total protein amount is determined by MemStain. The right side shows the SIRT3 membrane. Bands used for the respective figure are labeled in red.

1.3 Supplementary Tables

Supplementary Table 1, Clinical data of ALS patients

	Study cohort ALS		Study cohort HD	
	control	ALS	control	HD
All participants	16	12	7	15
participants ♂	12	10	1	7
participants ♀	4	2	6	8
Age all participants	61.1 ±13.3	62.3 ±12.1	54.5 ±16.3	65.6 ±13.7
Age ♂	58.9 ±12.2	65.30 ±10.9	55	67.1 ±9.2
Age ♀	67.5 ±16.1	47.0 ±1.4	54.5 ±17.9	64.9 ±16.6
Post mortem interval	52.4 ±22.0	78.0 ±54.3	28.4 ±10.4	21.6 ±9.4

Supplementary Table 1. Clinical characteristics of the post mortem patient samples and the respective controls, including, age and the post mortem interval (mean ±SD) for the ALS and HD study cohort. Of note, clinical data was only available for 15 out of 17 HD samples.

Supplementary Table 2. List of primers for murine targets

Target (mouse)	Gene	Forward 5’-3’	Reverse 5’-3’	NCBI NM number	Efficiency [%]	Temp. [°C]
Canonical ex1-ex2 peroxisome proliferative activated receptor, gamma, coactivator 1 alpha	*Canonical Ppargc1a*	AGAGTGTGCTGCTCTGGTTG	TTCCGATTGGTCGCTACACC	NM_008904.2	101.8	60
CNS-specific B1-B4 peroxisome proliferative activated receptor, gamma, coactivator 1 alpha	*CNS-specific Ppargc1a*	TACAACTACGGCTCCTCCTGG	TACCCTTCATCCATGGGGCTC	NM_008904.2	91.5	60
nuclear respiratory factor 1	*Nrf1*	GCTGCAGGTCCTGTGGGAAT	ACTCAAACACATGAGGCCGT	NM_001164226.1	110.4	60
catalase	*Cat*	CCTTCAAGTTGGTTAATGCAGA	CAAGTTTTTGATGCCCTGGT	NM_009804.2	83.8	60
superoxide dismutase 2	*Sod2*	CTGAAGTTCAATGGTGGGGGA	CCAGCAACTCTCCTTTGGGTT	NM_013671.3	91.5	60
transcription factor A	*Tfam*	CAAAGGATGATTCGGCTCAG	AAGCTGAATATATGCCTGCTTTTC	NM_009360.4	97.6	60

uncoupling protein 2	*Ucp2*	AGCCTGAGACCTCAAAGCAG	CCTTCAATCGGCAAGACG	NM_011671.4	96.5	60
sirtuin 3	*Sirt3*	GCTTGAGAGAGCATCTGGGAT	CCTGTCCGCCATCACATCAG	NM_022433	95.0	60
sirtuin 4	*Sirt4*	GGATGCATGCACAGAGTCCTG	CTCAGTGAGGAACACGTCGC	NM_001167691.1	94.9	60
sirtuin 5	*Sirt5*	TCTGGAAATCCACGGAACCT	ACTGGGATTCTGGCGTCTTG	NM_178848.3	104.8	62
hypoxanthine guanine phosphoribosyltransferase	*Hprt*	GGAGCGGTAGCACCTCCT	CTGGTTCATCATCGCTAATCAC	NM_013556.2	92.8	60
methionylaminopeptidase 1	*Metap1*	TGCGACTCGTGTGTAGGC	CTTCAGTAGTTACACCCGCTTTAAT	NM_175224.4	101.6	60
polymerase (RNA) II (DNA directed) polypeptide A	*Polr2a*	GCTGGGAGACATAGCACCA	TTACTCCCCTGCATGGTCTC	NM_001291068.1	94.8	60
TATA box binding protein	*Tbp*	GGCGGTTTGGCTAGGTTT	GGGTTATCTTCACACACCATGA	NM_013684.3	97.2	58,7
actin, beta	*Actb*	CCACCAGTTCGCCATGGAT	GGCTTTGCACATGCCGGAG	NM_007393.5	103.1	60
Ribosomal protein large P0	*Rplp0*	CGTCCTCGTTGGAGTGACAT	TAGTTGGACTTCCAGGTCGC	NM_007475.5	89.0	60

Supplementary Table 2. Murine primer sequences for the respective target genes are shown, indicating the target and the gene name. The NCBI number, the efficiency and the annealing temperature are added as well.

Supplementary Table 3. List of primers for human targets

**Target (human)**	**Gene**	**Forward 5'-3'**	**Reverse 5'-3'**	**NCBI NM number**	**Efficiency [%]**	**Temp. [°C]**
Canonical ex1-ex2 peroxisome proliferative activated receptor, gamma, coactivator 1 alpha	*Canonical Ppargc1a*	CGTGGGACATGTGCAACCAGG	GCTGTCTGTATCCAAGTCGT	NM_013261.4	117.3	60
CNS-specific B1-B4 peroxisome proliferative activated receptor, gamma, coactivator 1 alpha	*CNS-specific Ppargc1a*	TACAACTACGGCTCCTCCTGG	TACCCTTCATCCATGGGGCTC	NM_008904.2	85.3	60
sirtuin 3	*SIRT3*	GGGCTTGAGAGAGTGTCGG	GGAACCCTGTCTGCCATCAC	NM_012239.5	89.6	60
sirtuin 4	*SIRT4*	GTCCGTAGAGCTGTGAGAGAATG	ATCCAACGGCCTTTTGCTGA	NM_012240	103.5	60
sirtuin 5	*SIRT5*	GCTCGGCCAAGTTCAAGTATG	CCTCTGAAGGTCGGAACACC	NM_012241	106.4	60
polymerase (RNA) II (DNA directed) polypeptide A	*POLR2A*	TTGTGCAGGACACACTCACA	CAGGAGGTTCATCACTTCACC	NM_000937.4	105.4	60
TATA box binding protein	*TBP*	CCCATGACTCCCATGACC	TTTACAACCAAGATTCACTGTGG	NM_003194.4	94.0	60

Supplementary Table 3. Human primer sequences for the respective target genes are shown, indicating the target and the gene name. The NCBI number, the efficiency and the annealing temperature are added as well.

Supplementary Table 4. List of mouse primers for mtDNA copy number

**Target**	**Gene**	**Forward**	**Reverse**	**Temp. [°C]**
displacement loop	Dloop	AGGCATGAAAGGACAGCACA	GGTGATTGGGTTTTGCGGAC	60
beta-2 microglobulin	B2M	GCTCACACTGAATTCACCCC	CGGCCATACTGGCATGCTTA	60

Supplementary Table 4. Primer sequences for the determination of the mitochondrial DNA copy number in murine tissues and cells are shown with the respective annealing temperature.

Supplementary Table 5. *Sirt4* and *Sirt5* mRNA levels in SOD1(G93A) mice are stable in different tissues during the course of disease

**spinal cord**						
	60d		100d		130d	
	WT	SOD1	WT	SOD1	WT	SOD1
Sirt4	100 ±34.2	81.9±12.1	97.0±14.2	90.9±17.9	106.9±22.4	97.5±19.2
Sirt5	100±20.7	107.5±26.7	95.3±20.6	96.6±17.6	113.1±21.2	95.9±25.3
**brain stem**						
	60d		100d		130d	
	WT	SOD1	WT	SOD1	WT	SOD1
Sirt4	100±15.4	91.7±10.1	77.5±4.2	93.5±46.5	108.61±46.9	73.7±27.3
Sirt5	100±12.9	89.3±15.82	85.9±11.7	52.7±9.2**	84.0±19.6	58.3±19.5*
**hippocampus**						
	60d		100d		130d	
	WT	SOD1	WT	SOD1	WT	SOD1
Sirt4	100±39.6	93.2±32.3	90.3±8.4	96.3±21.2	100±20.5	90.3±19.3
Sirt5	100±22.3	96.4±17.8	95.8±14.6	104.2±21.5	105±17.82	88.3±24.7

Supplementary Table 5. *Sirt4* and *Sirt5* mRNA levels were determined in B6SJL-Tg(SOD1*G93A)1Gur/J (SOD1(G93A)) mice during the course of disease at 60 d (preonset), 100 d (onset) and at about 130 d (endstage) in the disease affected brain regions spinal cord, the brain stem and the control region hippocampus, n = 5 -8. mRNA levels were quantified by qPCR and normalized to TATA box binding protein (*Tbp*) and RNA polymerase 2 (*Polr2a*) for the spinal cord, to β-actin (*Actb*) and ribosomal protein large P0 (*Rplp0*) for the brain stem and to *Tbp*, *Polr2a* and *Actb* in for the hippocampus, relative to mean of wt 60 d old mice. The grouped data was analyzed using the two-way ANOVA test followed by Tukey’s multiple comparison test. Data are presented as mean ± SEM. Significance relative to 60d SOD1: *=P≤0.05, **=P≤0.01.

Supplementary Table 6. *Sirt4* and *Sirt5* mRNA levels in R6/2 mice are stable in most tissues during the course of disease

**striatum**						
	4we		8we		12we	
	WT	R6/2	WT	R6/2	WT	R6/2
Sirt4	100±10.43	90.13±14.8	107.2±14.6	134.6±17.6**	110.1±19.6	118.5±16.7
Sirt5	100±19.2	104.81±22.5	126.1±26.8	145.4±30.3	95.9±13.4	107.2±13.5
**cortex**						
	4we		8we		12we	
	WT	R6/2	WT	R6/2	WT	R6/2
Sirt4	100±13.3	85.6±11.4	101.9±12.2	76.7±8.4**	102.3±20.9	79.3±18.2*
Sirt5	100±24.4	95.0±26.2	105.7±21.1	105.9±19.2	110.9±25.8	104.6±39.0
**cerebellum**						
	4we		8we		12we	
	WT	R6/2	WT	R6/2	WT	R6/2
Sirt4	100±13.5	90.2±12.4	102.8±13.6	101.2±8.3	99.3±19.7	93.8±9.5
Sirt5	100±14.0	103.6±11.0	121±9.7	106.2±23.0	121.5±10.6	87.3±17.4**

Supplementary Table 6. *Sirt4* and *Sirt5* mRNA levels were determined in R6/2 mice during the course of disease at 4, 8 and 12 weeks of age in the disease affected brain regions spinal cord and cortex and the control region cerebellum, n = 5 -8. mRNA levels were measured using qPCR and data was normalized to TATA box binding protein (*Tbp*), RNA polymerase 2 (*Polr2a*) and β-actin (*Actb*) for striatum and cortex and to *Tbp*, *Actb* and methionylaminopeptidase 1 (*Metap1*) for the cerebellum, n = 5 – 9. The grouped data was analyzed using the two-way ANOVA test followed by Sidak’s multiple comparison test. Data are presented as mean ± SEM. Significance is shown relative to the WT control oft he respective age: *=P≤0.05, **=P≤0.01.
